# Analyzing the dynamical sensitivity and soliton solutions of time-fractional Schrödinger model with Beta derivative

**DOI:** 10.1038/s41598-024-58796-z

**Published:** 2024-04-09

**Authors:** Muhammad Nadeem, Fenglian Liu, Yahya Alsayaad

**Affiliations:** 1 School of Mathematics and Statistics, Qujing Normal University, Qujing, 655011 China; 2 Institute of Land & Resources and Sustainable Development, Yunnan University of Finance and Economics, Kunming, 650221 China; 3 Department of Physics, Hodeidah University, Al-Hudaydah, Yemen

**Keywords:** Nonlinear time-fractional coupled Schrödinger model, Modified Sardar sub-equation approach, Beta derivative, Soliton solutions, Sensitivity analysis, Applied optics, Optical physics

## Abstract

In physical domains, Beta derivatives are necessary to comprehend wave propagation across various nonlinear models. In this research work, the modified Sardar sub-equation approach is employed to find the soliton solutions of (1+1)-dimensional time-fractional coupled nonlinear Schrödinger model with Beta fractional derivative. These models are fundamental in real-world applications such as control systems, processing of signals, and fiber optic networks. By using this strategy, we are able to obtain various unique optical solutions, including combo, dark, bright, periodic, singular, and rational wave solutions. In addition, We address the sensitivity analysis of the proposed model to investigate the truth that it is extremely sensitive. These studies are novel and have not been performed before in relation to the nonlinear dynamic features of these solutions. We show these behaviors in 2-D, contour 3-D structures across the associated physical characteristics. Our results demonstrate that the proposed approach offers useful results for producing solutions of nonlinear fractional models in application of mathematics and wave propagation in fiber optics.

## Introduction

Fractional calculus (FC) is a field of mathematics that focuses on non-integer order derivatives and integrals. In recent years, various applications of FC have increased in the fields of physics, engineering, and applied mathematics. Numerous scholars have explored new theories and applications, like multiplicative fractional calculus and fuzzy logic to develop the models for real-world problems^1,2^. Novel kinds of inequalities are presented by FC and its applications with non-conformable fractional integrals. Numerous concepts have reported, including Riemann-Liouville, Atangana-Baleanu, Caputo-Fabrizio and conformable derivatives^3–5^. These fractional derivatives have applications in a wide range of scientific and technical disciplines. Wave motion in dispersive objects, viscoelastic material comprehension, fractal feature processing in signals, modeling biological systems with anomalous diffusion, and electromagnetic system understanding are some of its uses. They serve as fundamental concepts in the area of fractional calculus as well. When combined, these fractional derivatives provide an adaptable framework for explaining and understanding complex real-world phenomena. This versatility has led to advancements in several branches of signal processing, physics, biology, and materials research. Their significance originates from their capacity to describe systems with fractional-order effects, rendering them valuable tools for comprehending and addressing an extensive array of scientific and technical problems^6,7^.

A class of mathematical models known as nonlinear partial differential equations (NLPDEs) is needed to explain a broad range of natural events in quantum physics and wave propagation^8,9^. Beyond their mathematical elegance, NLPDEs have theoretical relevance because they provide a solid framework for modeling complex framework, mechanisms, and transfers in daily life challenges. Natural processes frequently exhibit nonlinear behavior; examples include soliton production, shock waves, and pattern self-organization. Because of their unpredictability, these occurrences call for intricate mathematical models that can depict the intricate relationships between numerous variables^10,11^.

A soliton is a specific type of solitary wave that acts as a particle and keeps its individuality if it interacts with another soliton. It is necessary to comprehend these individual waves to comprehend the dynamics of waves. Typically, a single soliton solution is referred to as a “solitary wave”. Solitons offer stable solutions for NLPDEs if the impacts of scattering and nonlinearity are completely balanced^12^. The concept of solitons has grown to be an exciting field of study and played a major impact on the recent developments in the telecom sector. Fibre optics has demonstrated the effectiveness of solitons in transmitting digital messages over vast distances without dispersing^13,14^. Various analytical and numerical techniques have been used for the solutions of nonlinear models, such as; the khater technique^15^, the unified technique^16^, the $\frac{G'}{G}$ technique^17^, the Hirota bilinear technique^18^, the extended $\tanh$-function scheme^19^, the $\textrm{F}$-expansion technique^20^, the modified simple equation method^21^, the homotopy perturbation technique^22^, the modified variational iteration technique^23^, the sine-Gordon expansion technique^24^ and the direct algebraic technique^25^ and so on^26–28^.

In this paper, we examine innovative soliton solutions and conduct sensitivity analysis to validate the sensitivity of the proposed model. In this work, the use of Beta derivatives to time-fractional coupled nonlinear Schrödinger (FCNLS) model is innovative since it allows for an additional complete analysis of solution spaces and discovers the previous unknown solution of frameworks. This study is designed as: Section “Beta fractional derivative”, discussed an overview of Beta fractional derivative and a physical significance of time FCNLS model. Section “Methodology of the MSSE approach” explains the idea of modified Sardar sub-equation (MSSE) approach. Section “Mathematical analysis” describes the extraction of soliton solutions. In Section “Dynamical system”, we discuss the features of dynamical system of the proposed model with sensitivity analysis. The results and discussions are presented in Section “Results and discussions”. The conclusion remarks of this study are discussed in Section “Conclusion remarks”.

## Beta fractional derivative

Recently, the concept of the Beta fractional derivative (FD) has been proposed by Atangana et al^29^., indicating an important development in study of mathematical derivatives. In particular, Beta FD offer greater capability in accurately predicting real-time phenomena compared to standard derivative. The main advantage of FD is its non-locality where it shows the impact of distant elements on the behavior of a system and adds fundamental value. Numerous fields, including dielectric polarization, viscoelasticity, electrical chemistry, processing of images, and magnetic systems, make extensive use of beta FD. Most of them appear in physics and engineering such as robotics, heat and mass transfer, biotechnology, and wave theory.

### Definition 2.1

The Beta FD of *g* with order ${\varsigma }~ \in ~(0,1]$ is expressed as

1 $$\begin{aligned} _{0}^{Q}{\mathfrak {D}}_{t}^{\varsigma }g(t) = \lim _{\gamma \rightarrow 0}\frac{g(t+\gamma (t+\frac{1}{\Gamma (\varsigma )}))-g(t)}{\gamma }, \quad \quad g~:~(0,\infty )~\rightarrow ~\mathbb {R}. \end{aligned}$$

In the open interval $(0,c),~c~>~0$, *g* is ${\varsigma }$-differentiable, and $\lim _{t \rightarrow 0^{+}} ({_{0}^Q}{\mathfrak {D}_t^{\varsigma }g(t)})$. Then

2 $$\begin{aligned} {_{0}^{Q}}\mathfrak {D}_{t}^{\varsigma }g(0)=\lim _{t \rightarrow 0^{+}} ({_{0}^Q}{\mathfrak {D}_t^{\varsigma }g(t)}). \end{aligned}$$

### Theorem 2.1

Let *g* is continuous at $t_0$, when $g~:~(0,\infty )~\rightarrow ~\mathbb {R}$ is $\varsigma$-differentiable for $t_0~>~0$, where $\varsigma ~\in ~(0,1].$

### Theorem 2.2

If $0~<~\varsigma ~\le ~1,$ *a*,  *b* $\in$ $\mathbb {R}$, $g,~u,~\varsigma$-differentiable, at a point $t~>~0$. Then

1. $${_{0}^{Q}}\mathfrak {D}_{t}^{\varsigma }(v_1 g+v_2 u)=v_1 ~{_{0}^{Q}}\mathfrak {D}_{t}^{\varsigma }(g)~+~v_2~ {_{0}^{Q}}\mathfrak {D}_{t}^{\varsigma }( u),$$ $$v_1,~v_2~\in ~\mathbb {R}.$$
2. ${_{0}^{Q}}\mathfrak {D}_{t}^{\varsigma }(\psi )=~0,$ in which $\psi$ is constant.
3. $${_{0}^{Q}}\mathfrak {D}_{t}^{\varsigma }(gu)=~g~{_{0}^{Q}}\mathfrak {D}_{t}^{\varsigma }(u)+~u~{_{0}^{Q}}\mathfrak {D}_{t}^{\varsigma }(g).$$
4. $${_{0}^{Q}}\mathfrak {D}_{t}^{\varsigma }(\frac{g}{u})=~\frac{u~{_{0}^{Q}}\mathfrak {D}_{t}^{\varsigma }(g)~-~g~{_{0}^{Q}}\mathfrak {D}_{t}^{\varsigma }(u)}{u^2}.$$
5. Assuming $\gamma ~=~(t+\frac{1}{\Gamma (\varsigma )})^{\varsigma -1}~f,~f~\rightarrow ~0~$ when $\gamma ~\rightarrow ~0,$ ${_{0}^{Q}}\mathfrak {D}_{t}^{\varsigma }(g(t))=(t+\frac{1}{\Gamma (\varsigma )})^{\varsigma -1}~ \frac{dg}{dt},$ with $\Phi =~\frac{l_{1}}{\varsigma }~(t+\frac{1}{\Gamma (\varsigma )})^{\varsigma }),$ as $l_{1}$ is constant,
6. $${_{0}^{Q}}\mathfrak {D}_{t}^{\varsigma }(\frac{g(t)}{u(x)})=l~\frac{dg(t)}{dt}.$$

### Mathematical model

The time FCNLS model in (1+1) dimension containing a fractional derivation (FD) of beta is as follows^30^

3 $$\begin{aligned} \mathfrak {D}_x \mathfrak {D}_t^\varsigma \mathcal {V}=\mathfrak {D}_{xx}\mathcal {V}+\frac{2}{1-\alpha ^{2}}|\mathcal {V}|^2\mathcal {V}+\mathcal {V}(\mathcal {R}-\mathcal {U}), \end{aligned}$$

4 $$\begin{aligned} \mathfrak {D}_t^{\varsigma } \mathcal {R}= \frac{-\mathfrak {D}_t^\varsigma (|\mathcal {V}|^2)}{1+\alpha }+(1+\alpha )\mathfrak {D}_{x} \mathcal {R}, \end{aligned}$$

5 $$\begin{aligned} \mathfrak {D}_t^\varsigma \mathcal {U}= \frac{-\mathfrak {D}_t^\varsigma (|\mathcal {V}|^2)}{1+\alpha }+(1-\alpha )\mathfrak {D}_{x} \mathcal {U}. \end{aligned}$$

In Beta FD, $\mathfrak {D}_{t}^\varsigma$ and $\mathfrak {D}_{x}^{2\varsigma }$ are real valued functions and $\mathcal {U}$ and $\mathcal {R}$ are complex functions. Nonlinear behavior in time FCNLS model causes the effects which extend over a simple linear composition of its parameter aspects. This nonlinearity enables exciting phenomena such as the generation of solitons, self-destructive, and change of energy among connected components. The study of the (1+1)-dimensional time FCNLS model with beta derivatives has gained increasing significance due to its numerous applications in various domains. Travelling waves in fractal media have been described via analytical solutions for a class of FCNLS models. The FCNLS model has been transformed into ordinary differential equations using new conformable fractional derivative techniques, which has made it easier to derive precise traveling wave solutions. Fractional dual-function and fractional Riccati methods have been used to find vector photonic soliton and periodic solutions for the FCNLS model. Fractional space-time derivatives have been the focus of investigations on the FCNLS model and new explosives^31,32^. Our paper proposes novel techniques to handle this complex problem using the MSSE approach and obtain the optical soliton solutions, time series, and sensitivity analysis^33^. This study has tremendous implications for engineering and scientific research since it provides insights into complex system behaviors and aids in the development of appropriate control systems. This research offer new avenues for research, especially because they have never been applied to the time FCNLS model. Our work follows a more general strategy, spanning a wide range of optical solutions and concentrating on specific solution types.

## Methodology of the MSSE approach

The modified Sardar sub-equation (MSSE) approach expands on the original Sardar sub-equation approach by incorporating additional variables and scenarios into the ansatz for solving nonlinear problems. This approach has been successfully applied to solve NLPDEs in many different areas of mathematics and science. The general form of NLPDEs is

6 $$\begin{aligned} W(\mathcal {V},~\mathfrak {D}_{x}^{\varsigma }\mathcal {V},~\mathfrak {D}_{x}^{2\varsigma }\mathcal {V},~\mathfrak {D}_{t}^{\varsigma }\mathcal {V},~\mathfrak {D}_{x}^{2\varsigma }\mathcal {V},...)=0. \end{aligned}$$

***Step-i:*** Utilizing the complex wave transformation into Eq. (4), we obtain

7 $$\begin{aligned} \mathcal {V}(x,t)=\mathcal {M}(\eta )e^{i \theta }, \quad \quad \mathcal {R}(x,t)=\mathcal {S}(\eta ), \quad \quad \mathcal {U}(x,t)=\mathcal {N}(\eta ) \nonumber \\ \eta =a x+\frac{b \left( t+\frac{1}{\Gamma (\varsigma )}\right) ^{\varsigma }}{\varsigma }, \quad \quad \theta =c x+\frac{l \left( t+\frac{1}{\Gamma (\varsigma )}\right) ^{\varsigma }}{\varsigma }, \end{aligned}$$

Thus, the nonlinear ordinary differential equations (NLODEs) is achieved as

8 $$\begin{aligned} {\mathcal {Q}(\mathcal {M},~\mathcal {M}^{'},~\mathcal {M}^{''},...)=0.} \end{aligned}$$

**Step 2.** According to the approach, the general solution of Eq. (8) is described in the following form

9 $$\begin{aligned} \mathcal {M}(\eta )=\mathcal {F}_{0}+\sum _{j=1}^{J}\mathcal {F}_{j}\mathcal {L}^{j}(\eta ),~~~~~~~~~\mathcal {F}_{j} \ne ~0, \end{aligned}$$

where $$\mathcal {M}=\mathcal {M}(\eta )$$ assures

10 $$\begin{aligned} \mathcal {L}'(\eta )^2=\omega _2 \mathcal {L}(\eta )^4+\omega _1 \mathcal {L}(\eta )^2+\omega _0, \end{aligned}$$

where $\omega _0\ne 1$, $\omega _1$ and $\omega _2\ne 0$ are integers. Compute the constants $\mathcal {F}_{0}$ and $\mathcal {F}_{1}$. Moreover, $\mathcal {F}_{j}$ is invertible, thus it can be zero. The values of *J* can be obtained by using balance principle. The cases to Eq. (10) are as follows.

**Case-1:**

- If $$\omega _0=0,~\omega _1>0~ \text {and}~ \omega _2 ~\ne 0$$, then
  11 $$\begin{aligned} \mathcal {L}_1(\eta )=\sqrt{-\frac{\omega _1}{\omega _2}} \text {sech}\left( \sqrt{\omega _1} (\eta +\tau )\right) . \end{aligned}$$
- If $$\omega _0=0,~\omega _1>0~\text {and}~\omega _2\ne 0$$, then
  12 $$\begin{aligned} \mathcal {L}_2(\eta )={\sqrt{\frac{\omega _1}{\omega _2}} \text {csch}\left( \sqrt{\omega _1} (\eta +\tau )\right) }. \end{aligned}$$

**Case-2:**

- For constants $k_{1}~\text {and}~k_{2}$, let $\omega _0=0,~\omega _1>0$ and $\omega _2=+4 k_1 k_2$, then
  13 $$\begin{aligned} \mathcal {L}_3(\eta )=\frac{4 k_1 \sqrt{\omega _1}}{\left( 4 k_1^2-\omega _2\right) \sinh \left( \sqrt{\omega _1} (\eta +\tau )\right) +\left( 4 k_1^2-\omega _2\right) \cosh \left( \sqrt{\omega _1} (\eta +\tau )\right) }. \end{aligned}$$

**Case-3:**

- For constants $E_{1}~\text {and}~E_{2}$, let $\omega _0=\frac{\omega _1^2}{4 \omega _2},\omega _1<0 ~\text {and}~ \omega _2>0$, then
  14 $$\begin{aligned} \mathcal {L}_4(\eta )=\sqrt{-\frac{\omega _1}{2 \omega _2}} \tanh \left( \sqrt{-\frac{\omega _1}{2}} (\eta +\tau )\right) . \end{aligned}$$
- For constants $E_{1}~\text {and}~E_{2}$, let $\omega _0=\frac{\omega _1^2}{4 \omega _2},\omega _1<0 ~\text {and}~ \omega _2>0$, then
  15 $$\begin{aligned} \mathcal {L}_5(\eta )=\sqrt{-\frac{\omega _1}{2 \omega _2}} \coth \left( \sqrt{-\frac{\omega _1}{2}} (\eta +\tau )\right) . \end{aligned}$$
- For constants $E_{1}~\text {and}~E_{2}$, let $\omega _0=\frac{\omega _1^2}{4 \omega _2},~\omega _1<0 ~\text {and}~ \omega _2>0$, then
  16 $$\begin{aligned} \mathcal {L}_6(\eta )=\sqrt{-\frac{\omega _1}{2 \omega _2}} \left( \tanh \left( \sqrt{-\frac{\omega _1}{2}} (\eta +\tau )\right) +i \text {sech}\left( \sqrt{-2 \omega _1} (\eta +\tau )\right) \right) . \end{aligned}$$
- For constants $E_{1}~\text {and}~E_{2}$, let $\omega _0=\frac{\omega _1^2}{4 \omega _2},\omega _1<0 ~\text {and}~ \omega _2>0$, then
  17 $$\begin{aligned} \mathcal {L}_7(\eta )=\sqrt{-\frac{\omega _1}{8 \omega _2}} \left( \tanh \left( \sqrt{-\frac{\omega _1}{8}} (\eta +\tau )\right) +\coth \left( \sqrt{-\frac{\omega _1}{8}} (\eta +\tau )\right) \right) . \end{aligned}$$
- For constants $E_{1}~\text {and}~E_{2}$, let $\omega _0=\frac{\omega _1^2}{4 \omega _2},~\omega _1<0 ~\text {and}~ \omega _2>0$, then
  18 $$\begin{aligned} \mathcal {L}_8(\eta )=\frac{\sqrt{-\frac{\omega _1}{2 \omega _2}} \left( \sqrt{{E}_1^2+{E}_2^2}-e_1 \cosh \left( \sqrt{-2 \omega _1} (\eta +\Psi )\right) \right) }{{E}_1 \sinh \left( \sqrt{-2 \omega _1} (\eta +\Psi )\right) +{E}_2}, \end{aligned}$$
  19 $$\begin{aligned} \mathcal {L}_9(\eta )=\frac{\sqrt{-\frac{\omega _1}{2 \omega _2}} \cosh \left( \sqrt{-2 \omega _1} (\eta +\tau )\right) }{\sinh \left( \sqrt{-2 \omega _1} (\eta +\tau )\right) +i}. \end{aligned}$$

**Case-4:**

- Let $$\omega _0=0,{\omega }_1<0~\text {and}~{\omega }_2\ne 0$$, then
  20 $$\begin{aligned} \mathcal {L}_{10}(\eta )=\sqrt{-\frac{\omega _1}{\omega _2}} \sec \left( \sqrt{-\omega _1} (\eta +\tau )\right) . \end{aligned}$$
- Let $$\omega _0=0,~{\omega }_1<0~\text {and}~{\omega }_2\ne 0$$, then
  21 $$\begin{aligned} \mathcal {L}_{11}(\eta )=\sqrt{-\frac{\omega _1}{\omega _2}} \csc \left( \sqrt{-\omega _1} (\eta +\tau )\right) . \end{aligned}$$

**Case-5:**

- Let $\omega _0=\frac{\omega _1^2}{4 \omega _2},~\omega _1>0$ and $\omega _2>0$ and  $E_1^2-E_2^2>0$, then
  22 $$\begin{aligned} \mathcal {L}_{12}(\eta )=\sqrt{-\frac{\omega _1}{2 \omega _2}} \tan \left( \sqrt{\frac{\omega _1}{2}} (\eta +\tau )\right) . \end{aligned}$$
- Let $\omega _0=\frac{\omega _1^2}{4 \omega _2},~\omega _1>0$ and $\omega _2>0$ and  ${E}_1^2-{E}_2^2>0$, then
  23 $$\begin{aligned} \mathcal {L}_{13}(\eta )=-\sqrt{-\frac{\omega _1}{2 \omega _2}} \cot \left( \sqrt{\frac{\omega _1}{2}} (\eta +\tau )\right) . \end{aligned}$$
- Let $\omega _0=\frac{\omega _1^2}{4 \omega _2},\omega _1>0$ and $\omega _2>0$ and  $E_1^2-E_2^2>0$, then
  24 $$\begin{aligned} \mathcal {L}_{14}(\eta )=-\sqrt{-\frac{\omega _1}{2\omega _2}} \left( \tan \left( \sqrt{2 \omega _1} (\eta +\tau )\right) -\sec \left( \sqrt{2 \omega _1} (\eta +\tau )\right) \right) . \end{aligned}$$
- Let $\omega _0=\frac{\omega _1^2}{4 \omega _2},~\omega _1>0$ and $\omega _2>0$ and  $E_1^2-E_2^2>0$, then
  25 $$\begin{aligned} \mathcal {L}_{15}(\eta )=\sqrt{-\frac{\omega _1}{8 \omega _2}} \left( \tan \left( \sqrt{\frac{\omega _1}{8}} (\eta +\tau )\right) -\cot \left( \sqrt{\frac{\omega _1}{8}} (\eta +\tau )\right) \right) . \end{aligned}$$
- Let $\omega _0=\frac{\omega _1^2}{4 \omega _2},~\omega _1>0$ and $\omega _2>0$ and  $E_1^2-E_2^2>0$, then
  26 $$\begin{aligned} \mathcal {L}_{16}(\eta )=\frac{\sqrt{-\frac{\omega _1}{2 \omega _2}} \left( \sqrt{E_1^2-E_2^2}-S_1 \cos \left( \sqrt{2 \omega _1} (\eta +\tau )\right) \right) }{E_2+S_1 \sin \left( \sqrt{2 \omega _1} (\eta +\tau )\right) }. \end{aligned}$$
  27 $$\begin{aligned} \mathcal {L}_{17}(\eta )=\frac{\sqrt{-\frac{\omega _1}{2 \omega _2}} \cos \left( \sqrt{2 \omega _1} (\eta +\tau )\right) }{\sin \left( \sqrt{2 \omega _1} (\eta +\tau )\right) -1}. \end{aligned}$$

**Case-6:**

- Let $$ \omega _0=0,~\omega _1>0,$$ then
  28 $$\begin{aligned} \mathcal {L}_{18}(\eta )=\frac{4 \omega _1 e^{\sqrt{\omega _1} (\eta +\tau )}}{e^{2 \sqrt{\omega _1} (\eta +\tau )}-4 \omega _1 \omega _2}. \end{aligned}$$
- Let $$ \omega _0=0,~\omega _1>0,$$ then
  29 $$\begin{aligned} \mathcal {L}_{19}(\eta )=\frac{4 \omega _1 e^{\sqrt{\omega _1} (\eta +\tau )}}{1-4 \omega _1 \omega _2 e^{2 \sqrt{\omega _1} (\eta +\tau )}}. \end{aligned}$$

**Case-7:**

- Let $$\omega _0=0,~\omega _1=0~\text {and}~\omega _2>0$$, then
  30 $$\begin{aligned} \mathcal {L}_{20}(\eta )=\frac{1}{\sqrt{\omega _2} (\eta +\tau )}. \end{aligned}$$
- Let $\omega _0=0,~\omega _1=0~$and$~\omega _2>0$, then
  31 $$\begin{aligned} \mathcal {L}_{21}(\eta )=\frac{i}{\sqrt{\omega _2} (\eta +\tau )}. \end{aligned}$$

**Step 3.** Put Eq. (9) into Eq. (8) and by using Eq. (10), the polynomial can be obtained as a power of $\mathcal {L}(\eta )$. **Step 4.** Assemble the similar parameters of $\mathcal {L}(\eta )$ and equating them to zero, we can obtain the algebraic system for $\mathcal {F}_{0},~ \mathcal {F}_{{j}}$     ($j=1,2,3,...$). **Step 5.** Finally, apply the Mathematica Software to the algebraic systems of equations to obtain the coefficients values. Putting these parameter values to Eq. (8), we get the solution of Eqs. (3, 4 and 5). The MSSE approach is a helpful tool for obtaining the precise results to NLPDEs, such as the (1+1)-dimensional FCNLS model. This method requires assuming an ansatz for results in terms of additional variables and a unique function, and then solving an algebraic system of equations to obtain the unknown constants.

## Mathematical analysis

This part concentrates on implementing our suggested approach to validate its effectiveness, performance, and reliability. Consequently, we obtain a soliton solution for the time-dimensional (1+1) FCNLS model. The Eq. (7) containing the complex transformation is employed. The Eq. (3) is now utilized to convert Eqs. (3), (4) and (5) into NLODEs. Consequently, the real and imaginary parts of NLODEs yields

32 $$\begin{aligned} \mathcal {M}''(\eta )+\left( \frac{c(c-l)}{a(b-a)} \right) \mathcal {M}(\eta )-\left( \frac{2}{a(b-a)(1-\alpha ^2}\right) \mathcal {M}^3(\eta )+\left( \frac{\mathcal {N}-\mathcal {S}}{a(b-a)}\right) \mathcal {M}(\eta )=0, \end{aligned}$$

and

33 $$\begin{aligned} la+bc+2ac=0, \end{aligned}$$

solve Eq. (33), we get

34 $$\begin{aligned} \frac{c-l}{b-a}=\frac{c}{a}, \end{aligned}$$

putting Eq. (34), into Eq. (32), we get

35 $$\begin{aligned} \mathcal {M}''(\eta )+\left( \frac{c^2}{a^2} \right) \mathcal {M}(\eta )-\left( \frac{2}{a(b-a)(1-\alpha ^2}\right) \mathcal {M}^3(\eta )+\left( \frac{\mathcal {N}-\mathcal {S}}{a(b-a)}\right) \mathcal {M}(\eta )=0. \end{aligned}$$

Inserting Eq. (7) into Eqs. (4 and 5), and then integrate, we get the following Eqs.

36 $$\begin{aligned} \mathcal {S}=\frac{-b \mathcal {M}^2}{(1+\alpha )(b-(1+\alpha )a)}, \end{aligned}$$

37 $$\begin{aligned} \mathcal {N}=\frac{b \mathcal {M}^2}{(1-\alpha )(b-(1-\alpha )a)}. \end{aligned}$$

Inserting Eqs. (36 and 37), into Eq. (32), acquire an ODE

38 $$\begin{aligned} \mathcal {M}''(\eta )+\left( \frac{c^2}{a^2} \right) \mathcal {M}(\eta )-\left( \frac{2}{a(b-a)(1-\alpha ^2}\right) \mathcal {M}^3(\eta )=0. \end{aligned}$$

Employing balance principle in Eq. (38), we obtain $j=1$. The precise results shown in Eq. (9) with ${J}=1$ is

39 $$\begin{aligned} \mathcal {M}(\eta )=\mathcal {F}_1 \mathcal {L}(\eta )+\mathcal {F}_0. \end{aligned}$$

On comparing the similar powers of $\mathcal {L}(\eta ))^{j}$ with $j=0,1,2,3,...$. We create a system of algebraic equations by combining Eq. (39) with Eq. (38), as well as Eq. (10). After evaluation, we obtain the following results presented below.

***Family-1:***

40 $$\begin{aligned} \begin{aligned} \left\{ \mathcal {F}_0\rightarrow 0,~~\mathcal {F}_1\rightarrow -\frac{\sqrt{\omega _2 \left( -\left( b^2 \omega _1+2 i b c \sqrt{\omega _1}+\left( \alpha ^2-1\right) c^2\right) \right) }}{\sqrt{\omega _1}},~~a\rightarrow -\frac{i c}{\sqrt{\omega _1}}\right\} . \end{aligned} \end{aligned}$$

It has been established that the aforementioned outcomes are satisfactory to Family 1.

41 $$\begin{aligned} \mathcal {V}_{1,1}(x,t)=-\frac{\sqrt{-\frac{\omega _1}{\omega _2}} \sqrt{\omega _2 \left( b^2 \left( -\omega _1\right) -2 i b c \sqrt{\omega _1}-\left( \alpha ^2-1\right) c^2\right) } {e^{i \theta }} \text {sech}\left( \sqrt{\omega _1} \left( \frac{b \left( t+\frac{1}{\Gamma (\varsigma )}\right) ^{\varsigma }}{\varsigma }-\frac{i c x}{\sqrt{\omega _1}}+\Psi \right) \right) }{\sqrt{\omega _1}}, \end{aligned}$$

- If $\omega _0=0,~\omega _1>0$ and $\omega _2 ~\ne ~0,$ we get

42 $$\begin{aligned} \mathcal {R}_{1,1}(x,t)= & {} \frac{-b \left( -\frac{\sqrt{-\frac{\omega _1}{\omega _2}} \sqrt{\omega _2 \left( b^2 \left( -\omega _1\right) -2 i b c \sqrt{\omega _1}-\left( \alpha ^2-1\right) c^2\right) } e^{i \left( c x+\frac{l \left( t+\frac{1}{\Gamma (\varsigma )}\right) ^{\varsigma }}{\varsigma }\right) } \text {sech}\left( \sqrt{\omega _1} \left( \frac{b \left( t+\frac{1}{\Gamma (\varsigma )}\right) ^{\varsigma }}{\varsigma }-\frac{i c x}{\sqrt{\omega _1}}+\Psi \right) \right) }{\sqrt{\omega _1}}\right) ^2}{(1+\alpha )(b-(1+\alpha )a)}, \end{aligned}$$

43 $$\begin{aligned} \mathcal {U}_{1,1}(x,t)= & {} \frac{b \left( -\frac{\sqrt{-\frac{\omega _1}{\omega _2}} \sqrt{\omega _2 \left( b^2 \left( -\omega _1\right) -2 i b c \sqrt{\omega _1}-\left( \alpha ^2-1\right) c^2\right) } e^{i \left( c x+\frac{l \left( t+\frac{1}{\Gamma (\varsigma )}\right) ^{\varsigma }}{\varsigma }\right) } \text {sech}\left( \sqrt{\omega _1} \left( \frac{b \left( t+\frac{1}{\Gamma (\varsigma )}\right) ^{\varsigma }}{\varsigma }-\frac{i c x}{\sqrt{\omega _1}}+\Psi \right) \right) }{\sqrt{\omega _1}}\right) ^2}{(1-\alpha )(b-(1-\alpha )a)}, \end{aligned}$$

44 $$\begin{aligned} \mathcal {V}_{1,2}(x,t)= & {} {-\frac{\sqrt{\frac{\omega _1}{\omega _2}} \sqrt{\omega _2 \left( b^2 \left( -\omega _1\right) -2 i b c \sqrt{\omega _1}-\left( \alpha ^2-1\right) c^2\right) } {e^{i \theta }} \text {csch}\left( \sqrt{\omega _1} \left( \frac{b \left( t+\frac{1}{\Gamma (\varsigma )}\right) ^{\varsigma }}{\varsigma }-\frac{i c x}{\sqrt{\omega _1}}+\Psi \right) \right) }{\sqrt{\omega _1}},} \end{aligned}$$

45 $$\begin{aligned} \mathcal {R}_{1,2}(x,t)= & {} \frac{-b \left( -\frac{\sqrt{\frac{\omega _1}{\omega _2}} \sqrt{\omega _2 \left( b^2 \left( -\omega _1\right) -2 i b c \sqrt{\omega _1}-\left( \alpha ^2-1\right) c^2\right) } e^{i \left( c x+\frac{l \left( t+\frac{1}{\Gamma (\varsigma )}\right) ^{\varsigma }}{\varsigma }\right) } \text {csch}\left( \sqrt{\omega _1} \left( \frac{b \left( t+\frac{1}{\Gamma (\varsigma )}\right) ^{\varsigma }}{\varsigma }-\frac{i c x}{\sqrt{\omega _1}}+\Psi \right) \right) }{\sqrt{\omega _1}}\right) ^2}{(1+\alpha )(b-(1+\alpha )a)}, \end{aligned}$$

46 $$\begin{aligned} \mathcal {U}_{1,2}(x,t)= & {} \frac{b \left( -\frac{\sqrt{\frac{\omega _1}{\omega _2}} \sqrt{\omega _2 \left( b^2 \left( -\omega _1\right) -2 i b c \sqrt{\omega _1}-\left( \alpha ^2-1\right) c^2\right) } e^{i \left( c x+\frac{l \left( t+\frac{1}{\Gamma (\varsigma )}\right) ^{\varsigma }}{\varsigma }\right) } \text {csch}\left( \sqrt{\omega _1} \left( \frac{b \left( t+\frac{1}{\Gamma (\varsigma )}\right) ^{\varsigma }}{\varsigma }-\frac{i c x}{\sqrt{\omega _1}}+\Psi \right) \right) }{\sqrt{\omega _1}}\right) ^2}{(1-\alpha )(b-(1-\alpha )a)}, \end{aligned}$$

- If $\omega _0=0,~\omega _1>0$ and $\omega _2 =4 K_1 K_2,$ we get

47 $$\begin{aligned} \mathcal {V}_{1,3}(x,t)= & {} -\frac{4 k_1 \sqrt{\omega _2 \left( b^2 \left( -\omega _1\right) -2 i b c \sqrt{\omega _1}-\left( \alpha ^2-1\right) c^2\right) } {e^{i \theta }}}{\left( 4 k_1^2-\omega _2\right) \cosh \left( \sqrt{\omega _1} \left( \frac{b \left( t+\frac{1}{\Gamma (\varsigma )}\right) ^{\varsigma }}{\varsigma }-\frac{i c x}{\sqrt{\omega _1}}+\Psi \right) \right) }\times (4 k_1 \sqrt{\omega _2 \left( b^2 \left( -\omega _1\right) -2 i b c \sqrt{\omega _1}- c^2\right) } {e^{i \theta }}) \nonumber \\{} & {} \frac{1}{+\left( 4 k_1^2-\omega _2\right) \sinh \left( \sqrt{\omega _1} \left( \frac{b \left( t+\frac{1}{\Gamma (\varsigma )}\right) ^{\varsigma }}{\varsigma }-\frac{i c x}{\sqrt{\omega _1}}+\Psi \right) \right) }, \end{aligned}$$

48 $$\begin{aligned} \mathcal {R}_{1,3}(x,t)= & {} \frac{-b \left( -\frac{4 k_1 \sqrt{\omega _2 \left( b^2 \left( -\omega _1\right) -2 i b c \sqrt{\omega _1}-\left( \alpha ^2-1\right) c^2\right) } {e^{i \theta }}}{\left( 4 k_1^2-\omega _2\right) \cosh \left( \sqrt{\omega _1} \left( \frac{b \left( t+\frac{1}{\Gamma (\varsigma )}\right) ^{\varsigma }}{\varsigma }-\frac{i c x}{\sqrt{\omega _1}}+\Psi \right) \right) +\left( 4 k_1^2-\omega _2\right) \sinh \left( \sqrt{\omega _1} \left( \frac{b \left( t+\frac{1}{\Gamma (\varsigma )}\right) ^{\varsigma }}{\varsigma }-\frac{i c x}{\sqrt{\omega _1}}+\Psi \right) \right) }\right) ^2}{(1+\alpha )(b-(1+\alpha )a)}, \end{aligned}$$

49 $$\begin{aligned} \mathcal {U}_{1,3}(x,t)= & {} \frac{b \left( -\frac{4 k_1 \sqrt{\omega _2 \left( b^2 \left( -\omega _1\right) -2 i b c \sqrt{\omega _1}-\left( \alpha ^2-1\right) c^2\right) } e^{i \left( c x+\frac{l \left( t+\frac{1}{\Gamma (\varsigma )}\right) ^{\varsigma }}{\varsigma }\right) }}{\left( 4 k_1^2-\omega _2\right) \cosh \left( \sqrt{\omega _1} \left( \frac{b \left( t+\frac{1}{\Gamma (\varsigma )}\right) ^{\varsigma }}{\varsigma }-\frac{i c x}{\sqrt{\omega _1}}+\Psi \right) \right) +\left( 4 k_1^2-\omega _2\right) \sinh \left( \sqrt{\omega _1} \left( \frac{b \left( t+\frac{1}{\Gamma (\varsigma )}\right) ^{\varsigma }}{\varsigma }-\frac{i c x}{\sqrt{\omega _1}}+\Psi \right) \right) }\right) ^2}{(1-\alpha )(b-(1-\alpha )a)}, \end{aligned}$$

- If $\omega _0=\frac{\omega _1^2}{4\omega _2},~\omega _1<0$ and $\omega _2 > ~0,$ we get

50 $$\begin{aligned} \mathcal {V}_{1,4}(x,t)= & {} -\frac{\sqrt{-\frac{\omega _1}{\omega _2}} \sqrt{\omega _2 \left( b^2 \left( -\omega _1\right) -2 i b c \sqrt{\omega _1}-\left( \alpha ^2-1\right) c^2\right) } {e^{i \theta }} \tanh \left( \frac{\sqrt{-\omega _1} \left( \frac{b \left( t+\frac{1}{\Gamma (\varsigma )}\right) ^{\varsigma }}{\varsigma }-\frac{i c x}{\sqrt{\omega _1}}+\Psi \right) }{\sqrt{2}}\right) }{\sqrt{2} \sqrt{\omega _1}}, \end{aligned}$$

51 $$\begin{aligned} \mathcal {R}_{1,4}(x,t)= & {} \frac{-b \left( -\frac{\sqrt{-\frac{\omega _1}{\omega _2}} \sqrt{\omega _2 \left( b^2 \left( -\omega _1\right) -2 i b c \sqrt{\omega _1}-\left( \alpha ^2-1\right) c^2\right) } e^{i \left( c x+\frac{l \left( t+\frac{1}{\Gamma (\varsigma )}\right) ^{\varsigma }}{\varsigma }\right) } \tanh \left( \frac{\sqrt{-\omega _1} \left( \frac{b \left( t+\frac{1}{\Gamma (\varsigma )}\right) ^{\varsigma }}{\varsigma }-\frac{i c x}{\sqrt{\omega _1}}+\Psi \right) }{\sqrt{2}}\right) }{\sqrt{2} \sqrt{\omega _1}} \right) ^2}{(1+\alpha )(b-(1+\alpha )a)}, \end{aligned}$$

52 $$\begin{aligned} \mathcal {U}_{1,4}(x,t)= & {} \frac{b \left( -\frac{\sqrt{-\frac{\omega _1}{\omega _2}} \sqrt{\omega _2 \left( b^2 \left( -\omega _1\right) -2 i b c \sqrt{\omega _1}-\left( \alpha ^2-1\right) c^2\right) } e^{i \left( c x+\frac{l \left( t+\frac{1}{\Gamma (\varsigma )}\right) ^{\varsigma }}{\varsigma }\right) } \tanh \left( \frac{\sqrt{-\omega _1} \left( \frac{b \left( t+\frac{1}{\Gamma (\varsigma )}\right) ^{\varsigma }}{\varsigma }-\frac{i c x}{\sqrt{\omega _1}}+\Psi \right) }{\sqrt{2}}\right) }{\sqrt{2} \sqrt{\omega _1}} \right) ^2}{(1-\alpha )(b-(1-\alpha )a)}, \end{aligned}$$

53 $$\begin{aligned} \mathcal {V}_{1,5}(x,t)= & {} -\frac{\sqrt{-\frac{\omega _1}{\omega _2}} \sqrt{\omega _2 \left( b^2 \left( -\omega _1\right) -2 i b c \sqrt{\omega _1}-\left( \alpha ^2-1\right) c^2\right) } {e^{i \theta }} \coth \left( \frac{\sqrt{-\omega _1} \left( \frac{b \left( t+\frac{1}{\Gamma (\varsigma )}\right) ^{\varsigma }}{\varsigma }-\frac{i c x}{\sqrt{\omega _1}}+\Psi \right) }{\sqrt{2}}\right) }{\sqrt{2} \sqrt{\omega _1}}, \end{aligned}$$

54 $$\begin{aligned} \mathcal {R}_{1,5}(x,t)= & {} \frac{-b \left( -\frac{\sqrt{-\frac{\omega _1}{\omega _2}} \sqrt{\omega _2 \left( b^2 \left( -\omega _1\right) -2 i b c \sqrt{\omega _1}-\left( \alpha ^2-1\right) c^2\right) } e^{i \left( c x+\frac{l \left( t+\frac{1}{\Gamma (\varsigma )}\right) ^{\varsigma }}{\varsigma }\right) } \coth \left( \frac{\sqrt{-\omega _1} \left( \frac{b \left( t+\frac{1}{\Gamma (\varsigma )}\right) ^{\varsigma }}{\varsigma }-\frac{i c x}{\sqrt{\omega _1}}+\Psi \right) }{\sqrt{2}}\right) }{\sqrt{2} \sqrt{\omega _1}} \right) ^2}{(1+\alpha )(b-(1+\alpha )a)}, \end{aligned}$$

55 $$\begin{aligned} \mathcal {U}_{1,5}(x,t)= & {} \frac{b \left( -\frac{\sqrt{-\frac{\omega _1}{\omega _2}} \sqrt{\omega _2 \left( b^2 \left( -\omega _1\right) -2 i b c \sqrt{\omega _1}-\left( \alpha ^2-1\right) c^2\right) } e^{i \left( c x+\frac{l \left( t+\frac{1}{\Gamma (\varsigma )}\right) ^{\varsigma }}{\varsigma }\right) } \coth \left( \frac{\sqrt{-\omega _1} \left( \frac{b \left( t+\frac{1}{\Gamma (\varsigma )}\right) ^{\varsigma }}{\varsigma }-\frac{i c x}{\sqrt{\omega _1}}+\Psi \right) }{\sqrt{2}}\right) }{\sqrt{2} \sqrt{\omega _1}} \right) ^2}{(1-\alpha )(b-(1-\alpha )a)}, \end{aligned}$$

56 $$\begin{aligned} \mathcal {V}_{1,6}(x,t)= & {} -\frac{\sqrt{-\frac{\omega _1}{\omega _2}} \sqrt{\omega _2 \left( b^2 c^2\right) } {e^{i \theta }} \left( \tanh \left( \sqrt{2} \sqrt{-\omega _1} \left( \frac{b \left( t+\frac{1}{\Gamma (\varsigma )}\right) ^{\varsigma }}{\varsigma }-\frac{i c x}{\sqrt{\omega _1}}+\Psi \right) \right) \right) }{\sqrt{2} \sqrt{\omega _1}} \nonumber \\{} & {} + \frac{+i \text {sech}\left( \sqrt{2} \sqrt{-\omega _1} \left( \frac{b \left( t+\frac{1}{\Gamma (\varsigma )}\right) ^{\varsigma }}{\varsigma }-\frac{i c x}{\sqrt{\omega _1}}+\Psi \right) \right) }{\sqrt{2} \sqrt{\omega _1}}, \end{aligned}$$

57 $$\begin{aligned} \mathcal {R}_{1,6}(x,t)= & {} \frac{-b \left( -\frac{\sqrt{-\frac{\omega _1}{\omega _2}} \sqrt{\omega _2 \left( b^2 c^2\right) } {e^{i \theta }} \left( \tanh \left( \sqrt{2} \left( \frac{b \left( t+\frac{1}{\Gamma (\varsigma )}\right) ^{\varsigma }}{\varsigma }-\frac{i c x}{\sqrt{\omega _1}}+\Psi \right) \right) +i \text {sech}\left( \sqrt{2} \sqrt{-\omega _1} \left( \frac{b \left( t+\frac{1}{\Gamma (\varsigma )}\right) ^{\varsigma }}{\varsigma }-\frac{i c x}{\sqrt{\omega _1}}+\Psi \right) \right) \right) }{\sqrt{2} \sqrt{\omega _1}}\right) ^2}{(1+\alpha )(b-(1+\alpha )a)}, \end{aligned}$$

58 $$\begin{aligned} \mathcal {U}_{1,6}(x,t)= & {} \frac{b \left( -\frac{\sqrt{-\frac{\omega _1}{\omega _2}} \sqrt{\omega _2 \left( b^2 c^2\right) } {e^{i \theta }} \left( \tanh \left( \sqrt{2} \sqrt{-\omega _1} \left( \frac{b \left( t+\frac{1}{\Gamma (\varsigma )}\right) ^{\varsigma }}{\varsigma }-\frac{i c x}{\sqrt{\omega _1}}+\Psi \right) \right) +i \text {sech}\left( \sqrt{2} \sqrt{-\omega _1} \left( \frac{b \left( t+\frac{1}{\Gamma (\varsigma )}\right) ^{\varsigma }}{\varsigma }-\frac{i c x}{\sqrt{\omega _1}}+\Psi \right) \right) \right) }{\sqrt{2} \sqrt{\omega _1}}\right) ^2}{(1-\alpha )(b-(1-\alpha )a)}, \end{aligned}$$

59 $$\begin{aligned} \mathcal {V}_{1,7}(x,t)= & {} -\frac{\sqrt{-\frac{\omega _1}{\omega _2}} \sqrt{\omega _2 \left( b^2 c^2\right) } {e^{i \theta }} \left( \tanh \left( \sqrt{2} \sqrt{-\omega _1} \left( \frac{b \left( t+\frac{1}{\Gamma (\varsigma )}\right) ^{\varsigma }}{\varsigma }-\frac{i c x}{\sqrt{\omega _1}}+\Psi \right) \right) \right) }{\sqrt{2} \sqrt{\omega _1}} \nonumber \\{} & {} + \frac{+i \text {coth}\left( \sqrt{2} \sqrt{-\omega _1} \left( \frac{b \left( t+\frac{1}{\Gamma (\varsigma )}\right) ^{\varsigma }}{\varsigma }-\frac{i c x}{\sqrt{\omega _1}}+\Psi \right) \right) }{\sqrt{2} \sqrt{\omega _1}}, \end{aligned}$$

60 $$\begin{aligned} \mathcal {R}_{1,7}(x,t)= & {} \frac{-b \left( -\frac{\sqrt{-\frac{\omega _1}{\omega _2}}\sqrt{\omega _2 \left( b^2 -\left( \alpha ^2-1\right) c^2\right) } {e^{i \theta }} \left( i \coth \left( \frac{\sqrt{-\omega _1} \left( \frac{b \left( t+\frac{1}{\Gamma (\varsigma )}\right) ^{\varsigma }}{\varsigma }+\Psi \right) }{2 \sqrt{2}}\right) +\tanh \left( \frac{\sqrt{-\omega _1} \left( \frac{b \left( t+\frac{1}{\Gamma (\varsigma )}\right) ^{\varsigma }}{\varsigma }-\frac{i c x}{\sqrt{\omega _1}}+\Psi \right) }{2 \sqrt{2}}\right) \right) }{2 \sqrt{2} \sqrt{\omega _1}}\right) ^2}{(1+\alpha )(b-(1+\alpha )a)}, \end{aligned}$$

61 $$\begin{aligned} \mathcal {U}_{1,7}(x,t)= & {} \frac{b \left( -\frac{\sqrt{-\frac{\omega _1}{\omega _2}} \sqrt{\omega _2 \left( b^2 -\left( \alpha ^2-1\right) c^2\right) } {e^{i \theta }} \left( i \coth \left( \frac{\sqrt{-\omega _1} \left( \frac{b \left( t+\frac{1}{\Gamma (\varsigma )}\right) ^{\varsigma }}{\varsigma }-\frac{i c x}{\sqrt{\omega _1}}+\Psi \right) }{2 \sqrt{2}}\right) +\tanh \left( \frac{\sqrt{-\omega _1} \left( \frac{b \left( t+\frac{1}{\Gamma (\varsigma )}\right) ^{\varsigma }}{\varsigma }-\frac{i c x}{\sqrt{\omega _1}}+\Psi \right) }{2 \sqrt{2}}\right) \right) }{2 \sqrt{2} \sqrt{\omega _1}} \right) ^2}{(1-\alpha )(b-(1-\alpha )a)}, \end{aligned}$$

62 $$\begin{aligned} \mathcal {V}_{1,8}(x,t)= & {} -\frac{\sqrt{-\frac{\omega _1}{\omega _2}} \sqrt{\omega _2 \left( b^2 \left( -\omega _1\right) c^2\right) } {e^{i \theta }} \left( \sqrt{{E}_1^2+{E}_2^2}-{E}_1 \cosh \left( \sqrt{2} \sqrt{-\omega _1} \left( \frac{b \left( t+\frac{1}{\Gamma (\varsigma )}\right) ^{\varsigma }}{\varsigma }-\frac{i c x}{\sqrt{\omega _1}}+\Psi \right) \right) \right) }{\sqrt{2} \sqrt{\omega _1} \left( {E}_2+{E}_1 \sinh \left( \sqrt{2} \sqrt{-\omega _1} \left( \frac{b \left( t+\frac{1}{\Gamma (\varsigma )}\right) ^{\varsigma }}{\varsigma }-\frac{i c x}{\sqrt{\omega _1}}+\Psi \right) \right) \right) }, \end{aligned}$$

63 $$\begin{aligned} \mathcal {R}_{1,8}(x,t)= & {} \frac{-b \left( -\frac{\sqrt{-\frac{\omega _1}{\omega _2}} \sqrt{\omega _2 \left( b^2 \left( -\omega _1\right) -2 i b c \sqrt{\omega _1}-\left( \alpha ^2-1\right) c^2\right) } {e^{i \theta }} \left( \sqrt{{E}_1^2+{E}_2^2}-{E}_1 \cosh \left( \sqrt{2} \sqrt{-\omega _1} \left( \frac{b \left( t+\frac{1}{\Gamma (\varsigma )}\right) ^{\varsigma }}{\varsigma }-\frac{i c x}{\sqrt{\omega _1}}+\Psi \right) \right) \right) }{\sqrt{2} \sqrt{\omega _1} \left( {E}_2+{E}_1 \sinh \left( \sqrt{2} \sqrt{-\omega _1} \left( \frac{b \left( t+\frac{1}{\Gamma (\varsigma )}\right) ^{\varsigma }}{\varsigma }-\frac{i c x}{\sqrt{\omega _1}}+\Psi \right) \right) \right) } \right) ^2}{(1+\alpha )(b-(1+\alpha )a)}, \end{aligned}$$

64 $$\begin{aligned} \mathcal {U}_{1,8}(x,t)= & {} \frac{b \left( -\frac{\sqrt{-\frac{\omega _1}{\omega _2}} \sqrt{\omega _2 \left( b^2 \left( -\omega _1\right) -2 i b c \sqrt{\omega _1}-\left( \alpha ^2-1\right) c^2\right) } {e^{i \theta }} \left( \sqrt{{E}_1^2+{E}_2^2}-{E}_1 \cosh \left( \sqrt{2} \sqrt{-\omega _1} \left( \frac{b \left( t+\frac{1}{\Gamma (\varsigma )}\right) ^{\varsigma }}{\varsigma }-\frac{i c x}{\sqrt{\omega _1}}+\Psi \right) \right) \right) }{\sqrt{2} \sqrt{\omega _1} \left( {E}_2+{E}_1 \sinh \left( \sqrt{2} \sqrt{-\omega _1} \left( \frac{b \left( t+\frac{1}{\Gamma (\varsigma )}\right) ^{\varsigma }}{\varsigma }-\frac{i c x}{\sqrt{\omega _1}}+\Psi \right) \right) \right) }\right) ^2}{(1-\alpha )(b-(1-\alpha )a)}, \end{aligned}$$

65 $$\begin{aligned} \mathcal {V}_{1,9}(x,t)= & {} -\frac{\sqrt{-\frac{\omega _1}{\omega _2}} \sqrt{\omega _2 \left( b^2 \left( -\omega _1\right) -2 i b c \sqrt{\omega _1}-\left( \alpha ^2-1\right) c^2\right) } {e^{i \theta }} \cosh \left( \sqrt{2} \sqrt{-\omega _1} \left( \frac{b \left( t+\frac{1}{\Gamma (\varsigma )}\right) ^{\varsigma }}{\varsigma }-\frac{i c x}{\sqrt{\omega _1}}+\Psi \right) \right) }{\sqrt{2} \sqrt{\omega _1} \left( \sinh \left( \sqrt{2} \sqrt{-\omega _1} \left( \frac{b \left( t+\frac{1}{\Gamma (\varsigma )}\right) ^{\varsigma }}{\varsigma }-\frac{i c x}{\sqrt{\omega _1}}+\Psi \right) \right) +i\right) }, \end{aligned}$$

66 $$\begin{aligned} \mathcal {R}_{1,9}(x,t)= & {} \frac{-b \left( -\frac{\sqrt{-\frac{\omega _1}{\omega _2}} \sqrt{\omega _2 \left( b^2 \left( -\omega _1\right) -2 i b c \sqrt{\omega _1}-\left( \alpha ^2-1\right) c^2\right) } e^{i \left( c x+\frac{l \left( t+\frac{1}{\Gamma (\varsigma )}\right) ^{\varsigma }}{\varsigma }\right) } \cosh \left( \sqrt{2} \sqrt{-\omega _1} \left( \frac{b \left( t+\frac{1}{\Gamma (\varsigma )}\right) ^{\varsigma }}{\varsigma }-\frac{i c x}{\sqrt{\omega _1}}+\Psi \right) \right) }{\sqrt{2} \sqrt{\omega _1} \left( \sinh \left( \sqrt{2} \sqrt{-\omega _1} \left( \frac{b \left( t+\frac{1}{\Gamma (\varsigma )}\right) ^{\varsigma }}{\varsigma }-\frac{i c x}{\sqrt{\omega _1}}+\Psi \right) \right) +i\right) }\right) ^2}{(1+\alpha )(b-(1+\alpha )a)}, \end{aligned}$$

67 $$\begin{aligned} \mathcal {U}_{1,9}(x,t)= & {} \frac{b \left( -\frac{\sqrt{-\frac{\omega _1}{\omega _2}} \sqrt{\omega _2 \left( b^2 \left( -\omega _1\right) -2 i b c \sqrt{\omega _1}-\left( \alpha ^2-1\right) c^2\right) } e^{i \left( c x+\frac{l \left( t+\frac{1}{\Gamma (\varsigma )}\right) ^{\varsigma }}{\varsigma }\right) } \cosh \left( \sqrt{2} \sqrt{-\omega _1} \left( \frac{b \left( t+\frac{1}{\Gamma (\varsigma )}\right) ^{\varsigma }}{\varsigma }-\frac{i c x}{\sqrt{\omega _1}}+\Psi \right) \right) }{\sqrt{2} \sqrt{\omega _1} \left( \sinh \left( \sqrt{2} \sqrt{-\omega _1} \left( \frac{b \left( t+\frac{1}{\Gamma (\varsigma )}\right) ^{\varsigma }}{\varsigma }-\frac{i c x}{\sqrt{\omega _1}}+\Psi \right) \right) +i\right) } \right) ^2}{(1-\alpha )(b-(1-\alpha )a)}, \end{aligned}$$

- If $\omega _0=0,~\omega _1<0$ and $\omega _2 ~\ne ~0,$ we get

68 $$\begin{aligned} \mathcal {V}_{1,10}(x,t)= & {} -\frac{\sqrt{-\frac{\omega _1}{\omega _2}} \sqrt{\omega _2 \left( b^2 \left( -\omega _1\right) -2 i b c \sqrt{\omega _1}-\left( \alpha ^2-1\right) c^2\right) } {e^{i \theta }} \sec \left( \sqrt{-\omega _1} \left( \frac{b \left( t+\frac{1}{\Gamma (\varsigma )}\right) ^{\varsigma }}{\varsigma }-\frac{i c x}{\sqrt{\omega _1}}+\Psi \right) \right) }{\sqrt{\omega _1}}, \end{aligned}$$

69 $$\begin{aligned} \mathcal {R}_{1,10}(x,t)= & {} \frac{-b \left( -\frac{\sqrt{-\frac{\omega _1}{\omega _2}} \sqrt{\omega _2 \left( b^2 \left( -\omega _1\right) -2 i b c \sqrt{\omega _1}-\left( \alpha ^2-1\right) c^2\right) } e^{i \left( c x+\frac{l \left( t+\frac{1}{\Gamma (\varsigma )}\right) ^{\varsigma }}{\varsigma }\right) } \sec \left( \sqrt{-\omega _1} \left( \frac{b \left( t+\frac{1}{\Gamma (\varsigma )}\right) ^{\varsigma }}{\varsigma }-\frac{i c x}{\sqrt{\omega _1}}+\Psi \right) \right) }{\sqrt{\omega _1}}\right) ^2}{(1+\alpha )(b-(1+\alpha )a)}, \end{aligned}$$

70 $$\begin{aligned} \mathcal {U}_{1,10}(x,t)= & {} \frac{b \left( -\frac{\sqrt{-\frac{\omega _1}{\omega _2}} \sqrt{\omega _2 \left( b^2 \left( -\omega _1\right) -2 i b c \sqrt{\omega _1}-\left( \alpha ^2-1\right) c^2\right) } e^{i \left( c x+\frac{l \left( t+\frac{1}{\Gamma (\varsigma )}\right) ^{\varsigma }}{\varsigma }\right) } \sec \left( \sqrt{-\omega _1} \left( \frac{b \left( t+\frac{1}{\Gamma (\varsigma )}\right) ^{\varsigma }}{\varsigma }-\frac{i c x}{\sqrt{\omega _1}}+\Psi \right) \right) }{\sqrt{\omega _1}} \right) ^2}{(1-\alpha )(b-(1-\alpha )a)}, \end{aligned}$$

71 $$\begin{aligned} \mathcal {V}_{1,11}(x,t)= & {} -\frac{\sqrt{-\frac{\omega _1}{\omega _2}} \sqrt{\omega _2 \left( b^2 \left( -\omega _1\right) -2 i b c \sqrt{\omega _1}-\left( \alpha ^2-1\right) c^2\right) } {e^{i \theta }} \csc \left( \sqrt{-\omega _1} \left( \frac{b \left( t+\frac{1}{\Gamma (\varsigma )}\right) ^{\varsigma }}{\varsigma }-\frac{i c x}{\sqrt{\omega _1}}+\Psi \right) \right) }{\sqrt{\omega _1}}, \end{aligned}$$

72 $$\begin{aligned} \mathcal {R}_{1,11}(x,t)= & {} \frac{-b \left( -\frac{\sqrt{-\frac{\omega _1}{\omega _2}} \sqrt{\omega _2 \left( b^2 \left( -\omega _1\right) -2 i b c \sqrt{\omega _1}-\left( \alpha ^2-1\right) c^2\right) } e^{i \left( c x+\frac{l \left( t+\frac{1}{\Gamma (\varsigma )}\right) ^{\varsigma }}{\varsigma }\right) } \csc \left( \sqrt{-\omega _1} \left( \frac{b \left( t+\frac{1}{\Gamma (\varsigma )}\right) ^{\varsigma }}{\varsigma }-\frac{i c x}{\sqrt{\omega _1}}+\Psi \right) \right) }{\sqrt{\omega _1}} \right) ^2}{(1+\alpha )(b-(1+\alpha )a)}, \end{aligned}$$

73 $$\begin{aligned} \mathcal {U}_{1,11}(x,t)= & {} \frac{b \left( -\frac{\sqrt{-\frac{\omega _1}{\omega _2}} \sqrt{\omega _2 \left( b^2 \left( -\omega _1\right) -2 i b c \sqrt{\omega _1}-\left( \alpha ^2-1\right) c^2\right) } e^{i \left( c x+\frac{l \left( t+\frac{1}{\Gamma (\varsigma )}\right) ^{\varsigma }}{\varsigma }\right) } \csc \left( \sqrt{-\omega _1} \left( \frac{b \left( t+\frac{1}{\Gamma (\varsigma )}\right) ^{\varsigma }}{\varsigma }-\frac{i c x}{\sqrt{\omega _1}}+\Psi \right) \right) }{\sqrt{\omega _1}}\right) ^2}{(1-\alpha )(b-(1-\alpha )a)}, \end{aligned}$$

- If $\omega _0=\dfrac{\omega _1^2}{4\omega _2},~\omega _1>0$ and $\omega _2 > ~0,$ we get

74 $$\begin{aligned} \mathcal {V}_{1,12}(x,t)= & {} -\frac{\sqrt{\frac{\omega _1}{\omega _2}} \sqrt{\omega _2 \left( b^2 \left( -\omega _1\right) -2 i b c \sqrt{\omega _1}-\left( \alpha ^2-1\right) c^2\right) } e^{i \left( c x+\frac{l \left( t+\frac{1}{\Gamma (\varsigma )}\right) ^{\varsigma }}{\varsigma }\right) } \tan \left( \frac{\sqrt{\omega _1} \left( \frac{b \left( t+\frac{1}{\Gamma (\varsigma )}\right) ^{\varsigma }}{\varsigma }-\frac{i c x}{\sqrt{\omega _1}}+\Psi \right) }{\sqrt{2}}\right) }{\sqrt{2} \sqrt{\omega _1}}, \end{aligned}$$

75 $$\begin{aligned} \mathcal {R}_{1,12}(x,t)= & {} \frac{-b \left( -\frac{\sqrt{\frac{\omega _1}{\omega _2}} \sqrt{\omega _2 \left( b^2 \left( -\omega _1\right) -2 i b c \sqrt{\omega _1}-\left( \alpha ^2-1\right) c^2\right) } e^{i \left( c x+\frac{l \left( t+\frac{1}{\Gamma (\varsigma )}\right) ^{\varsigma }}{\varsigma }\right) } \tan \left( \frac{\sqrt{\omega _1} \left( \frac{b \left( t+\frac{1}{\Gamma (\varsigma )}\right) ^{\varsigma }}{\varsigma }-\frac{i c x}{\sqrt{\omega _1}}+\Psi \right) }{\sqrt{2}}\right) }{\sqrt{2} \sqrt{\omega _1}} \right) ^2}{(1+\alpha )(b-(1+\alpha )a)}, \end{aligned}$$

76 $$\begin{aligned} \mathcal {U}_{1,12}(x,t)= & {} \frac{b \left( -\frac{\sqrt{\frac{\omega _1}{\omega _2}} \sqrt{\omega _2 \left( b^2 \left( -\omega _1\right) -2 i b c \sqrt{\omega _1}-\left( \alpha ^2-1\right) c^2\right) } e^{i \left( c x+\frac{l \left( t+\frac{1}{\Gamma (\varsigma )}\right) ^{\varsigma }}{\varsigma }\right) } \tan \left( \frac{\sqrt{\omega _1} \left( \frac{b \left( t+\frac{1}{\Gamma (\varsigma )}\right) ^{\varsigma }}{\varsigma }-\frac{i c x}{\sqrt{\omega _1}}+\Psi \right) }{\sqrt{2}}\right) }{\sqrt{2} \sqrt{\omega _1}} \right) ^2}{(1-\alpha )(b-(1-\alpha )a)}, \end{aligned}$$

77 $$\begin{aligned} \mathcal {V}_{1,13}(x,t)= & {} -\frac{\sqrt{\frac{\omega _1}{\omega _2}} \sqrt{\omega _2 \left( b^2 \left( -\omega _1\right) -2 i b c \sqrt{\omega _1}-\left( \alpha ^2-1\right) c^2\right) } e^{i \left( c x+\frac{l \left( t+\frac{1}{\Gamma (\varsigma )}\right) ^{\varsigma }}{\varsigma }\right) } \cot \left( \frac{\sqrt{\omega _1} \left( \frac{b \left( t+\frac{1}{\Gamma (\varsigma )}\right) ^{\varsigma }}{\varsigma }-\frac{i c x}{\sqrt{\omega _1}}+\Psi \right) }{\sqrt{2}}\right) }{\sqrt{2} \sqrt{\omega _1}}, \end{aligned}$$

78 $$\begin{aligned} \mathcal {R}_{1,13}(x,t)= & {} \frac{-b \left( -\frac{\sqrt{\frac{\omega _1}{\omega _2}} \sqrt{\omega _2 \left( b^2 \left( -\omega _1\right) -2 i b c \sqrt{\omega _1}-\left( \alpha ^2-1\right) c^2\right) } e^{i \left( c x+\frac{l \left( t+\frac{1}{\Gamma (\varsigma )}\right) ^{\varsigma }}{\varsigma }\right) } \cot \left( \frac{\sqrt{\omega _1} \left( \frac{b \left( t+\frac{1}{\Gamma (\varsigma )}\right) ^{\varsigma }}{\varsigma }-\frac{i c x}{\sqrt{\omega _1}}+\Psi \right) }{\sqrt{2}}\right) }{\sqrt{2} \sqrt{\omega _1}} \right) ^2}{(1+\alpha )(b-(1+\alpha )a)}, \end{aligned}$$

79 $$\begin{aligned} \mathcal {U}_{1,13}(x,t)= & {} \frac{b \left( -\frac{\sqrt{\frac{\omega _1}{\omega _2}} \sqrt{\omega _2 \left( b^2 \left( -\omega _1\right) -2 i b c \sqrt{\omega _1}-\left( \alpha ^2-1\right) c^2\right) } e^{i \left( c x+\frac{l \left( t+\frac{1}{\Gamma (\varsigma )}\right) ^{\varsigma }}{\varsigma }\right) } \cot \left( \frac{\sqrt{\omega _1} \left( \frac{b \left( t+\frac{1}{\Gamma (\varsigma )}\right) ^{\varsigma }}{\varsigma }-\frac{i c x}{\sqrt{\omega _1}}+\Psi \right) }{\sqrt{2}}\right) }{\sqrt{2} \sqrt{\omega _1}} \right) ^2}{(1-\alpha )(b-(1-\alpha )a)}, \end{aligned}$$

80 $$\begin{aligned} \mathcal {V}_{1,14}(x,t)= & {} \frac{\sqrt{\frac{\omega _1}{\omega _2}} \sqrt{\omega _2} {e^{i \theta }} \left( \tan \left( \sqrt{2} \sqrt{\omega _1} \left( \frac{b \left( t+\frac{1}{\Gamma (\varsigma )}\right) ^{\varsigma }}{\varsigma }-\frac{i c x}{\sqrt{\omega _1}}+\Psi \right) \right) -\sec \left( \sqrt{2} \sqrt{\omega _1} \left( \frac{b \left( t+\frac{1}{\Gamma (\varsigma )}\right) ^{\varsigma }}{\varsigma }-\frac{i c x}{\sqrt{\omega _1}}+\Psi \right) \right) \right) }{\sqrt{2} \sqrt{\omega _1}}, \end{aligned}$$

81 $$\begin{aligned} \mathcal {R}_{1,14}(x,t)= & {} \frac{-b \left( \frac{\sqrt{\frac{\omega _1}{\omega _2}} \sqrt{\omega _2 \left( b^2 \left( \alpha ^2-1\right) c^2\right) } {e^{i \theta }} \left( \tan \left( \sqrt{2} \sqrt{\omega _1} \left( \frac{b \left( t+\frac{1}{\Gamma (\varsigma )}\right) ^{\varsigma }}{\varsigma }-\frac{i c x}{\sqrt{\omega _1}}+\Psi \right) \right) -\sec \left( \sqrt{2} \sqrt{\omega _1} \left( \frac{b \left( t+\frac{1}{\Gamma (\varsigma )}\right) ^{\varsigma }}{\varsigma }-\frac{i c x}{\sqrt{\omega _1}}+\Psi \right) \right) \right) }{\sqrt{2} \sqrt{\omega _1}}\right) ^2}{(1+\alpha )(b-(1+\alpha )a)}, \end{aligned}$$

82 $$\begin{aligned} \mathcal {U}_{1,14}(x,t)= & {} \frac{b \left( \frac{\sqrt{\frac{\omega _1}{\omega _2}} \sqrt{\omega _2 \left( b^2 \left( \alpha ^2-1\right) c^2\right) } {e^{i \theta }} \left( \tan \left( \sqrt{2} \sqrt{\omega _1} \left( \frac{b \left( t+\frac{1}{\Gamma (\varsigma )}\right) ^{\varsigma }}{\varsigma }-\frac{i c x}{\sqrt{\omega _1}}+\Psi \right) \right) -\sec \left( \sqrt{2} \sqrt{\omega _1} \left( \frac{b \left( t+\frac{1}{\Gamma (\varsigma )}\right) ^{\varsigma }}{\varsigma }-\frac{i c x}{\sqrt{\omega _1}}+\Psi \right) \right) \right) }{\sqrt{2} \sqrt{\omega _1}} \right) ^2}{(1-\alpha )(b-(1-\alpha )a)}, \end{aligned}$$

83 $$\begin{aligned} \mathcal {V}_{1,15}(x,t)= & {} -\frac{\sqrt{\frac{\omega _1}{\omega _2}} \sqrt{\omega _2 \left( b^2 -\left( \alpha \right) c^2\right) } {e^{i \theta }} \left( \tan \left( \frac{\sqrt{\omega _1} \left( \frac{b \left( t+\frac{1}{\Gamma (\varsigma )}\right) ^{\varsigma }}{\varsigma }-\frac{i c x}{\sqrt{\omega _1}}+\Psi \right) }{2 \sqrt{2}}\right) -\cot \left( \frac{\sqrt{\omega _1} \left( \frac{b \left( t+\frac{1}{\Gamma (\varsigma )}\right) ^{\varsigma }}{\varsigma }-\frac{i c x}{\sqrt{\omega _1}}+\Psi \right) }{2 \sqrt{2}}\right) \right) }{2 \sqrt{2} \sqrt{\omega _1}}, \end{aligned}$$

84 $$\begin{aligned} \mathcal {R}_{1,15}(x,t)= & {} \frac{-b \left( -\frac{\sqrt{\frac{\omega _1}{\omega _2}} \sqrt{\omega _2 \left( b^2 \sqrt{\omega _1}-\left( \alpha ^2-1\right) c^2\right) } {e^{i \theta }} \left( \tan \left( \frac{\sqrt{\omega _1} \left( \frac{b \left( t+\frac{1}{\Gamma (\varsigma )}\right) ^{\varsigma }}{\varsigma }-\frac{i c x}{\sqrt{\omega _1}}+\Psi \right) }{2 \sqrt{2}}\right) -\cot \left( \frac{\sqrt{\omega _1} \left( \frac{b \left( t+\frac{1}{\Gamma (\varsigma )}\right) ^{\varsigma }}{\varsigma }-\frac{i c x}{\sqrt{\omega _1}}+\Psi \right) }{2 \sqrt{2}}\right) \right) }{2 \sqrt{2} \sqrt{\omega _1}} \right) ^2}{(1+\alpha )(b-(1+\alpha )a)}, \end{aligned}$$

85 $$\begin{aligned} \mathcal {U}_{1,15}(x,t)= & {} \frac{b \left( -\frac{\sqrt{\frac{\omega _1}{\omega _2}} \sqrt{\omega _2 \left( b^2 \sqrt{\omega _1}-\left( \alpha ^2-1\right) c^2\right) } {e^{i \theta }} \left( \tan \left( \frac{\sqrt{\omega _1} \left( \frac{b \left( t+\frac{1}{\Gamma (\varsigma )}\right) ^{\varsigma }}{\varsigma }-\frac{i c x}{\sqrt{\omega _1}}+\Psi \right) }{2 \sqrt{2}}\right) -\cot \left( \frac{\sqrt{\omega _1} \left( \frac{b \left( t+\frac{1}{\Gamma (\varsigma )}\right) ^{\varsigma }}{\varsigma }-\frac{i c x}{\sqrt{\omega _1}}+\Psi \right) }{2 \sqrt{2}}\right) \right) }{2 \sqrt{2} \sqrt{\omega _1}} \right) ^2}{(1-\alpha )(b-(1-\alpha )a)}, \end{aligned}$$

86 $$\begin{aligned} \mathcal {V}_{1,16}(x,t)= & {} -\frac{\sqrt{\frac{\omega _1}{\omega _2}} \sqrt{\omega _2 \left( b^2 \left( -\omega _1\right) \left( \alpha ^2-1\right) c^2\right) } {e^{i \theta }} \left( \sqrt{e_1^2-e_2^2}-e_1 \cos \left( \sqrt{2} \sqrt{\omega _1} \left( \frac{b \left( t+\frac{1}{\Gamma (\varsigma )}\right) ^{\varsigma }}{\varsigma }-\frac{i c x}{\sqrt{\omega _1}}+\Psi \right) \right) \right) }{\sqrt{2} \sqrt{\omega _1} \left( e_2+e_1 \sin \left( \sqrt{2} \sqrt{\omega _1} \left( \frac{b \left( t+\frac{1}{\Gamma (\varsigma )}\right) ^{\varsigma }}{\varsigma }-\frac{i c x}{\sqrt{\omega _1}}+\Psi \right) \right) \right) }, \end{aligned}$$

87 $$\begin{aligned} \mathcal {R}_{1,16}(x,t)= & {} \frac{-b \left( -\frac{\sqrt{\frac{\omega _1}{\omega _2}} \sqrt{\omega _2 \left( b^2 \left( -\omega _1\right) -2 i b c \sqrt{\omega _1}-\left( \alpha ^2-1\right) c^2\right) } {e^{i \theta }} \left( \sqrt{e_1^2-e_2^2}-e_1 \cos \left( \sqrt{2} \sqrt{\omega _1} \left( \frac{b \left( t+\frac{1}{\Gamma (\varsigma )}\right) ^{\varsigma }}{\varsigma }-\frac{i c x}{\sqrt{\omega _1}}+\Psi \right) \right) \right) }{\sqrt{2} \sqrt{\omega _1} \left( e_2+e_1 \sin \left( \sqrt{2} \sqrt{\omega _1} \left( \frac{b \left( t+\frac{1}{\Gamma (\varsigma )}\right) ^{\varsigma }}{\varsigma }-\frac{i c x}{\sqrt{\omega _1}}+\Psi \right) \right) \right) }\right) ^2}{(1+\alpha )(b-(1+\alpha )a)}, \end{aligned}$$

88 $$\begin{aligned} \mathcal {U}_{1,16}(x,t)= & {} \frac{b \left( -\frac{\sqrt{\frac{\omega _1}{\omega _2}} \sqrt{\omega _2 \left( b^2 \left( -\omega _1\right) -2 i b c \sqrt{\omega _1}-\left( \alpha ^2-1\right) c^2\right) } {e^{i \theta }} \left( \sqrt{e_1^2-e_2^2}-e_1 \cos \left( \sqrt{2} \sqrt{\omega _1} \left( \frac{b \left( t+\frac{1}{\Gamma (\varsigma )}\right) ^{\varsigma }}{\varsigma }-\frac{i c x}{\sqrt{\omega _1}}+\Psi \right) \right) \right) }{\sqrt{2} \sqrt{\omega _1} \left( e_2+e_1 \sin \left( \sqrt{2} \sqrt{\omega _1} \left( \frac{b \left( t+\frac{1}{\Gamma (\varsigma )}\right) ^{\varsigma }}{\varsigma }-\frac{i c x}{\sqrt{\omega _1}}+\Psi \right) \right) \right) } \right) ^2}{(1-\alpha )(b-(1-\alpha )a)}, \end{aligned}$$

89 $$\begin{aligned} \mathcal {V}_{1,17}(x,t)= & {} -\frac{\sqrt{\frac{\omega _1}{\omega _2}} \sqrt{\omega _2 \left( b^2 \left( -\omega _1\right) -2 i b c \sqrt{\omega _1}-\left( \alpha ^2-1\right) c^2\right) } {e^{i \theta }} \cot \left( \sqrt{2} \sqrt{\omega _1} \left( \frac{b \left( t+\frac{1}{\Gamma (\varsigma )}\right) ^{\varsigma }}{\varsigma }-\frac{i c x}{\sqrt{\omega _1}}+\Psi \right) \right) }{\sqrt{2} \sqrt{\omega _1}}, \end{aligned}$$

90 $$\begin{aligned} \mathcal {R}_{1,17}(x,t)= & {} \frac{-b \left( -\frac{\sqrt{\frac{\omega _1}{\omega _2}} \sqrt{\omega _2 \left( b^2 \left( -\omega _1\right) -2 i b c \sqrt{\omega _1}-\left( \alpha ^2-1\right) c^2\right) } e^{i \left( c x+\frac{l \left( t+\frac{1}{\Gamma (\varsigma )}\right) ^{\varsigma }}{\varsigma }\right) } \cot \left( \sqrt{2} \sqrt{\omega _1} \left( \frac{b \left( t+\frac{1}{\Gamma (\varsigma )}\right) ^{\varsigma }}{\varsigma }-\frac{i c x}{\sqrt{\omega _1}}+\Psi \right) \right) }{\sqrt{2} \sqrt{\omega _1}} \right) ^2}{(1+\alpha )(b-(1+\alpha )a)}, \end{aligned}$$

91 $$\begin{aligned} \mathcal {U}_{1,17}(x,t)= & {} \frac{b \left( -\frac{\sqrt{\frac{\omega _1}{\omega _2}} \sqrt{\omega _2 \left( b^2 \left( -\omega _1\right) -2 i b c \sqrt{\omega _1}-\left( \alpha ^2-1\right) c^2\right) } e^{i \left( c x+\frac{l \left( t+\frac{1}{\Gamma (\varsigma )}\right) ^{\varsigma }}{\varsigma }\right) } \cot \left( \sqrt{2} \sqrt{\omega _1} \left( \frac{b \left( t+\frac{1}{\Gamma (\varsigma )}\right) ^{\varsigma }}{\varsigma }-\frac{i c x}{\sqrt{\omega _1}}+\Psi \right) \right) }{\sqrt{2} \sqrt{\omega _1}} \right) ^2}{(1-\alpha )(b-(1-\alpha )a)}, \end{aligned}$$

- If $\omega _0=0$ and $\omega _1>0$, we get

92 $$\begin{aligned} \mathcal {V}_{1,18}(x,t)= & {} -\frac{4 \sqrt{\omega _1} \sqrt{\omega _2 \left( b^2 \left( -\omega _1\right) -2 i b c \sqrt{\omega _1}-\left( \alpha ^2-1\right) c^2\right) } \exp \left( \sqrt{\omega _1} \left( \frac{b \left( t+\frac{1}{\Gamma (\varsigma )}\right) ^{\varsigma }}{\varsigma }-\frac{i c x}{\sqrt{\omega _1}}+\Psi \right) \right) }{-4 \omega _1 \omega _2+\exp \left( 2 \sqrt{\omega _1} \left( \frac{b \left( t+\frac{1}{\Gamma (\varsigma )}\right) ^{\varsigma }}{\varsigma }-\frac{i c x}{\sqrt{\omega _1}}+\Psi \right) \right) } \nonumber \\{} & {} \frac{+i \left( c x+\frac{l \left( t+\frac{1}{\Gamma (\varsigma )}\right) ^{\varsigma }}{\varsigma }\right) }{-4 \omega _1 \omega _2+\exp \left( 2 \sqrt{\omega _1} \left( \frac{b \left( t+\frac{1}{\Gamma (\varsigma )}\right) ^{\varsigma }}{\varsigma }-\frac{i c x}{\sqrt{\omega _1}}+\Psi \right) \right) }, \end{aligned}$$

93 $$\begin{aligned} \mathcal {R}_{1,18}(x,t)= & {} \frac{-b \left( -\frac{4 \sqrt{\omega _1} \sqrt{\omega _2 \left( b^2 \left( -\omega _1\right) -2 i b c \sqrt{\omega _1}-\left( \alpha ^2-1\right) c^2\right) } \exp \left( \sqrt{\omega _1} \left( \frac{b \left( t+\frac{1}{\Gamma (\varsigma )}\right) ^{\varsigma }}{\varsigma }-\frac{i c x}{\sqrt{\omega _1}}+\Psi \right) +i \left( c x+\frac{l \left( t+\frac{1}{\Gamma (\varsigma )}\right) ^{\varsigma }}{\varsigma }\right) \right) }{-4 \omega _1 \omega _2+\exp \left( 2 \sqrt{\omega _1} \left( \frac{b \left( t+\frac{1}{\Gamma (\varsigma )}\right) ^{\varsigma }}{\varsigma }-\frac{i c x}{\sqrt{\omega _1}}+\Psi \right) \right) } \right) ^2}{(1+\alpha )(b-(1+\alpha )a)}, \end{aligned}$$

94 $$\begin{aligned} \mathcal {U}_{1,18}(x,t)= & {} \frac{b \left( -\frac{4 \sqrt{\omega _1} \sqrt{\omega _2 \left( b^2 \left( -\omega _1\right) -2 i b c \sqrt{\omega _1}-\left( \alpha ^2-1\right) c^2\right) } \exp \left( \sqrt{\omega _1} \left( \frac{b \left( t+\frac{1}{\Gamma (\varsigma )}\right) ^{\varsigma }}{\varsigma }-\frac{i c x}{\sqrt{\omega _1}}+\Psi \right) +i \left( c x+\frac{l \left( t+\frac{1}{\Gamma (\varsigma )}\right) ^{\varsigma }}{\varsigma }\right) \right) }{-4 \omega _1 \omega _2+\exp \left( 2 \sqrt{\omega _1} \left( \frac{b \left( t+\frac{1}{\Gamma (\varsigma )}\right) ^{\varsigma }}{\varsigma }-\frac{i c x}{\sqrt{\omega _1}}+\Psi \right) \right) }\right) ^2}{(1-\alpha )(b-(1-\alpha )a)}, \end{aligned}$$

95 $$\begin{aligned} \mathcal {V}_{1,19}(x,t)= & {} -\frac{4 \sqrt{\omega _1} \sqrt{\omega _2 \left( b^2 \left( -\omega _1\right) -2 i b c \sqrt{\omega _1}-\left( \alpha ^2-1\right) c^2\right) } \exp \left( \sqrt{\omega _1} \left( \frac{b \left( t+\frac{1}{\Gamma (\varsigma )}\right) ^{\varsigma }}{\varsigma }-\frac{i c x}{\sqrt{\omega _1}}+\Psi \right) \right) }{1-4 \omega _1 \omega _2 \exp \left( 2 \sqrt{\omega _1} \left( \frac{b \left( t+\frac{1}{\Gamma (\varsigma )}\right) ^{\varsigma }}{\varsigma }-\frac{i c x}{\sqrt{\omega _1}}+\Psi \right) \right) } \nonumber \\{} & {} \times \frac{+i \left( c x+\frac{l \left( t+\frac{1}{\Gamma (\varsigma )}\right) ^{\varsigma }}{\varsigma }\right) }{1-4 \omega _1 \omega _2 \exp \left( 2 \sqrt{\omega _1} \left( \frac{b \left( t+\frac{1}{\Gamma (\varsigma )}\right) ^{\varsigma }}{\varsigma }-\frac{i c x}{\sqrt{\omega _1}}+\Psi \right) \right) }, \end{aligned}$$

96 $$\begin{aligned} \mathcal {R}_{1,19}(x,t)= & {} \frac{-b \left( -\frac{4 \sqrt{\omega _1} \sqrt{\omega _2 \left( b^2 \left( -\omega _1\right) -2 i b c \sqrt{\omega _1}-\left( \alpha ^2-1\right) c^2\right) } \exp \left( \sqrt{\omega _1} \left( \frac{b \left( t+\frac{1}{\Gamma (\varsigma )}\right) ^{\varsigma }}{\varsigma }-\frac{i c x}{\sqrt{\omega _1}}+\Psi \right) +i \left( c x+\frac{l \left( t+\frac{1}{\Gamma (\varsigma )}\right) ^{\varsigma }}{\varsigma }\right) \right) }{1-4 \omega _1 \omega _2 \exp \left( 2 \sqrt{\omega _1} \left( \frac{b \left( t+\frac{1}{\Gamma (\varsigma )}\right) ^{\varsigma }}{\varsigma }-\frac{i c x}{\sqrt{\omega _1}}+\Psi \right) \right) } \right) ^2}{(1+\alpha )(b-(1+\alpha )a)}, \end{aligned}$$

97 $$\begin{aligned} \mathcal {U}_{1,19}(x,t)= & {} \frac{b \left( -\frac{4 \sqrt{\omega _1} \sqrt{\omega _2 \left( b^2 \left( -\omega _1\right) -2 i b c \sqrt{\omega _1}-\left( \alpha ^2-1\right) c^2\right) } \exp \left( \sqrt{\omega _1} \left( \frac{b \left( t+\frac{1}{\Gamma (\varsigma )}\right) ^{\varsigma }}{\varsigma }-\frac{i c x}{\sqrt{\omega _1}}+\Psi \right) +i \left( c x+\frac{l \left( t+\frac{1}{\Gamma (\varsigma )}\right) ^{\varsigma }}{\varsigma }\right) \right) }{1-4 \omega _1 \omega _2 \exp \left( 2 \sqrt{\omega _1} \left( \frac{b \left( t+\frac{1}{\Gamma (\varsigma )}\right) ^{\varsigma }}{\varsigma }-\frac{i c x}{\sqrt{\omega _1}}+\Psi \right) \right) }\right) ^2}{(1-\alpha )(b-(1-\alpha )a)}, \end{aligned}$$

- If $\omega _0=0$, $\omega _1=0$ and $\omega _2>0$, we get

98 $$\begin{aligned} \mathcal {V}_{1,20}(x,t)= & {} -\frac{\sqrt{\omega _2 \left( b^2 \left( -\omega _1\right) -2 i b c \sqrt{\omega _1}-\left( \alpha ^2-1\right) c^2\right) } e^{i \left( c x+\frac{l \left( t+\frac{1}{\Gamma (\varsigma )}\right) ^{\varsigma }}{\varsigma }\right) }}{\sqrt{\omega _1} \sqrt{\omega _2} \left( \frac{b \left( t+\frac{1}{\Gamma (\varsigma )}\right) ^{\varsigma }}{\varsigma }-\frac{i c x}{\sqrt{\omega _1}}+\Psi \right) }, \end{aligned}$$

99 $$\begin{aligned} \mathcal {R}_{1,20}(x,t)= & {} \frac{-b \left( -\frac{\sqrt{\omega _2 \left( b^2 \left( -\omega _1\right) -2 i b c \sqrt{\omega _1}-\left( \alpha ^2-1\right) c^2\right) } e^{i \left( c x+\frac{l \left( t+\frac{1}{\Gamma (\varsigma )}\right) ^{\varsigma }}{\varsigma }\right) }}{\sqrt{\omega _1} \sqrt{\omega _2} \left( \frac{b \left( t+\frac{1}{\Gamma (\varsigma )}\right) ^{\varsigma }}{\varsigma }-\frac{i c x}{\sqrt{\omega _1}}+\Psi \right) }\right) ^2}{(1+\alpha )(b-(1+\alpha )a)}, \end{aligned}$$

100 $$\begin{aligned} \mathcal {U}_{1,20}(x,t)= & {} \frac{b \left( -\frac{\sqrt{\omega _2 \left( b^2 \left( -\omega _1\right) -2 i b c \sqrt{\omega _1}-\left( \alpha ^2-1\right) c^2\right) } e^{i \left( c x+\frac{l \left( t+\frac{1}{\Gamma (\varsigma )}\right) ^{\varsigma }}{\varsigma }\right) }}{\sqrt{\omega _1} \sqrt{\omega _2} \left( \frac{b \left( t+\frac{1}{\Gamma (\varsigma )}\right) ^{\varsigma }}{\varsigma }-\frac{i c x}{\sqrt{\omega _1}}+\Psi \right) } \right) ^2}{(1-\alpha )(b-(1-\alpha )a)}, \end{aligned}$$

101 $$\begin{aligned} \mathcal {V}_{1,21}(x,t)= & {} -\frac{i \sqrt{\omega _2 \left( b^2 \left( -\omega _1\right) -2 i b c \sqrt{\omega _1}-\left( \alpha ^2-1\right) c^2\right) } e^{i \left( c x+\frac{l \left( t+\frac{1}{\Gamma (\varsigma )}\right) ^{\varsigma }}{\varsigma }\right) }}{\sqrt{\omega _1} \sqrt{-\omega _2} \left( \frac{b \left( t+\frac{1}{\Gamma (\varsigma )}\right) ^{\varsigma }}{\varsigma }-\frac{i c x}{\sqrt{\omega _1}}+\Psi \right) }, \end{aligned}$$

102 $$\begin{aligned} \mathcal {R}_{1,21}(x,t)= & {} \frac{-b \left( -\frac{i \sqrt{\omega _2 \left( b^2 \left( -\omega _1\right) -2 i b c \sqrt{\omega _1}-\left( \alpha ^2-1\right) c^2\right) } e^{i \left( c x+\frac{l \left( t+\frac{1}{\Gamma (\varsigma )}\right) ^{\varsigma }}{\varsigma }\right) }}{\sqrt{\omega _1} \sqrt{-\omega _2} \left( \frac{b \left( t+\frac{1}{\Gamma (\varsigma )}\right) ^{\varsigma }}{\varsigma }-\frac{i c x}{\sqrt{\omega _1}}+\Psi \right) } \right) ^2}{(1+\alpha )(b-(1+\alpha )a)}, \end{aligned}$$

103 $$\begin{aligned} \mathcal {U}_{1,21}(x,t)= & {} \frac{b \left( -\frac{i \sqrt{\omega _2 \left( b^2 \left( -\omega _1\right) -2 i b c \sqrt{\omega _1}-\left( \alpha ^2-1\right) c^2\right) } e^{i \left( c x+\frac{l \left( t+\frac{1}{\Gamma (\varsigma )}\right) ^{\varsigma }}{\varsigma }\right) }}{\sqrt{\omega _1} \sqrt{-\omega _2} \left( \frac{b \left( t+\frac{1}{\Gamma (\varsigma )}\right) ^{\varsigma }}{\varsigma }-\frac{i c x}{\sqrt{\omega _1}}+\Psi \right) }\right) ^2}{(1-\alpha )(b-(1-\alpha )a)}. \end{aligned}$$

## Dynamical system

A system of dynamic is applied to describe the temporal dependency of a location of the point within its connecting area^34^. It is a collection of criteria that outline how parameters shift through time, the sensitivity of the concerning model, and how a system evolves. Dynamic systems are widely used in many fields, such as mathematics, biological sciences, chemistry, science and engineering, and financial studies. Population dynamics, chemical reactions, engineering problems, and the Schrödinger model are among the applications for these systems. Complex instances that predict the effects of changes in a range of sectors necessitate a deep understanding of dynamic applications and structures. The Eq. (38) can be turned into a dynamical framework after utilizing a particular modification. Now, consider

104 $$\begin{aligned} \mathcal {M}'(\eta )=P, \quad \mathcal {M}''(\eta )=P', \end{aligned}$$

After applying the aforementioned transformation to Eq. (104), we obtained the dynamical system that follows

105 $$\begin{aligned}{} & {} P=B_1,\nonumber \\{} & {} P'=B_2=-\frac{c^2}{a^2}\mathcal {M}(\eta )+\left( \frac{2}{\alpha ^2 a^2-(b-a)^2}\right) \mathcal {M}^3(\eta ). \end{aligned}$$

We can obtain the sensitivity analysis of the concerning framework utilizing the dynamical system of Eq. (105) by applying varied time-variant and initial conditions.

### Sensitivity analysis

Sensitivity analysis is a mathematical approach to assessing the effect of alternations in a framework of variables on its output. It is crucial to understand the capacity and reliability of dynamic structures. This analysis is commonly applied to investigate the changes in variables or configuration that affect the performance of systems in several types of disciplines, including energy, ecological structures, and dynamical framework^35^. The graphical representations of sensitivity analysis under appropriate parameter values and initial conditions are shown in Figs. (14-17).

**Figure 1:**
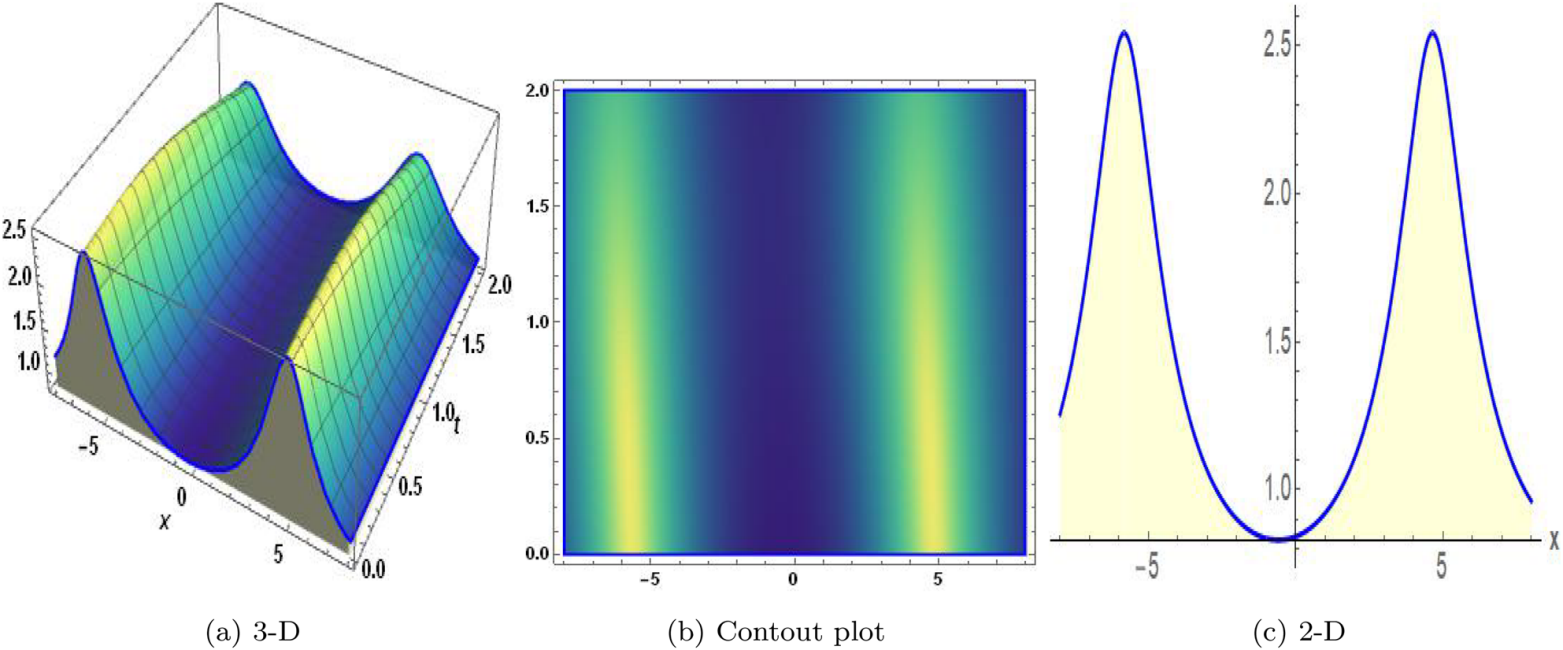
The values of parameters are $\omega _1 =1.5,~\omega _2=3.2,~b =2.03,~c=1.32,~l=1.23, {\varsigma =0.4}~ {\text{and}} ~\Psi _{2}=1.23,$ displays the graphical representation of Eq. (41).

**Figure 2:**
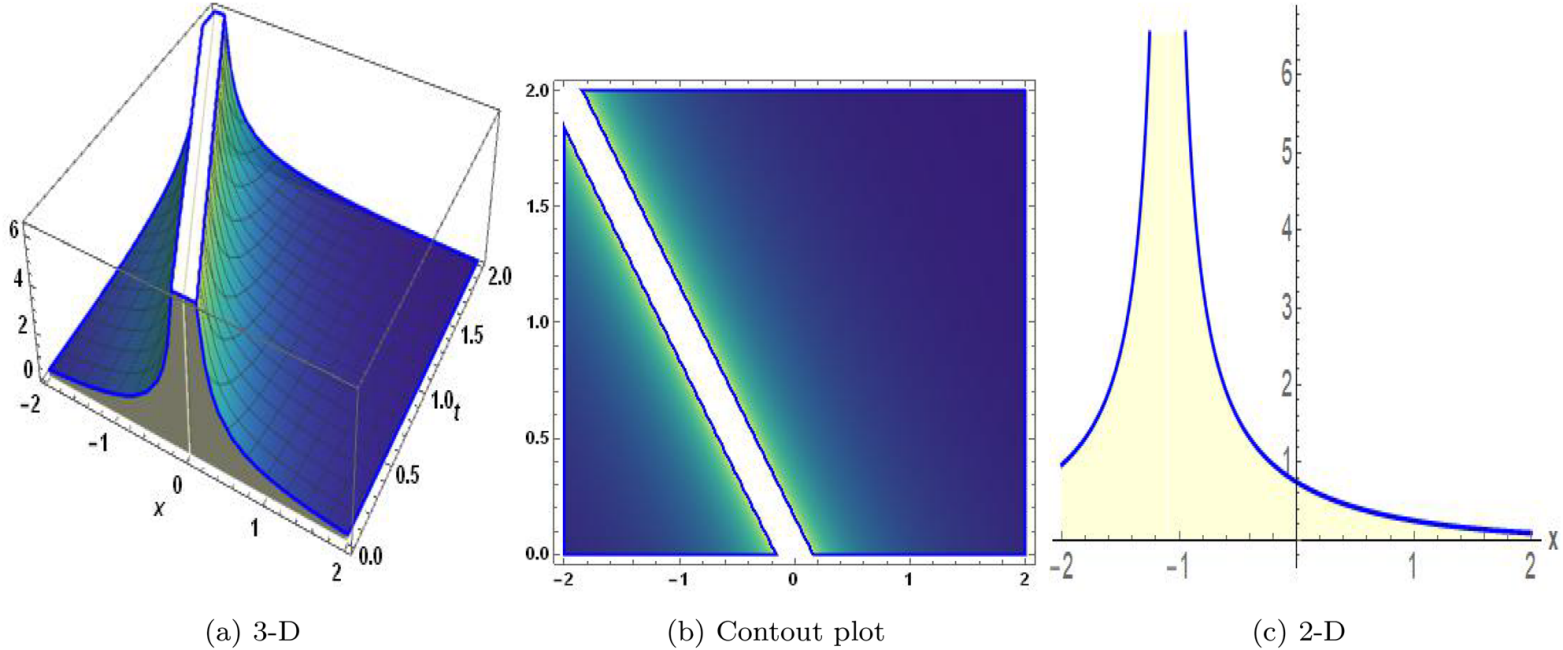
The values of parameters are $\omega _1 =1.5,~\omega _2=3.2,~b =2.03,~c=1.32,~l=1.23,{\varsigma =0.14}~ {\text{and}} ~\Psi _{2}=1.23,$ displays the graphical representation of Eq. (44).

**Figure 3:**
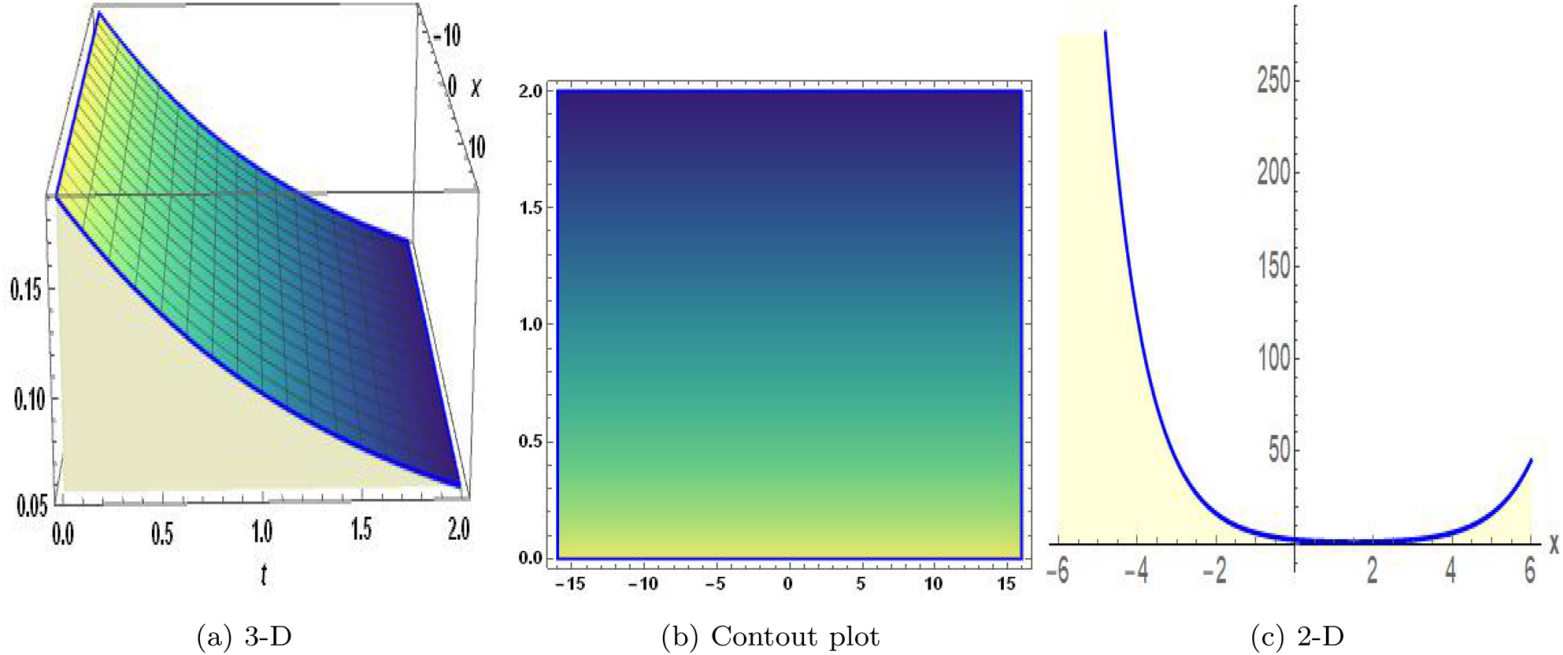
The values of parameters are $\omega _1 =1.5,~\omega _2=3.2,~b =2.03,~c=1.32,~l=1.23,~{\varsigma =1.4}~ {\text{and}} ~\Psi _{2}=1.23,$ displays the graphical representation of Eq. (47).

**Figure 4:**
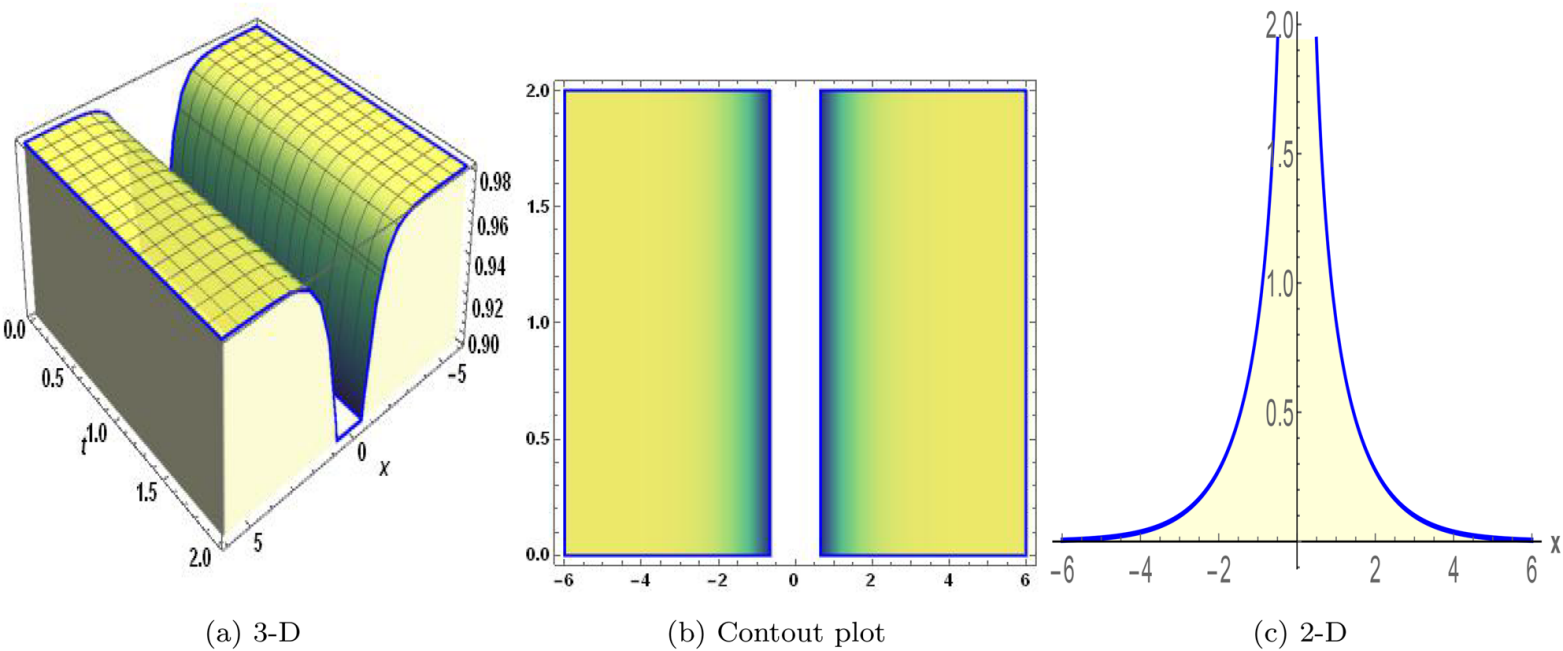
The values of parameters are $\omega _1 =1.5,~\omega _2=3.2,~b =2.03,~c=1.32,~l=1.23,~{\varsigma =2.4}~ {\text{and}} ~\Psi _{2}=1.23,$ displays the graphical representation of Eq. (50).

**Figure 5:**
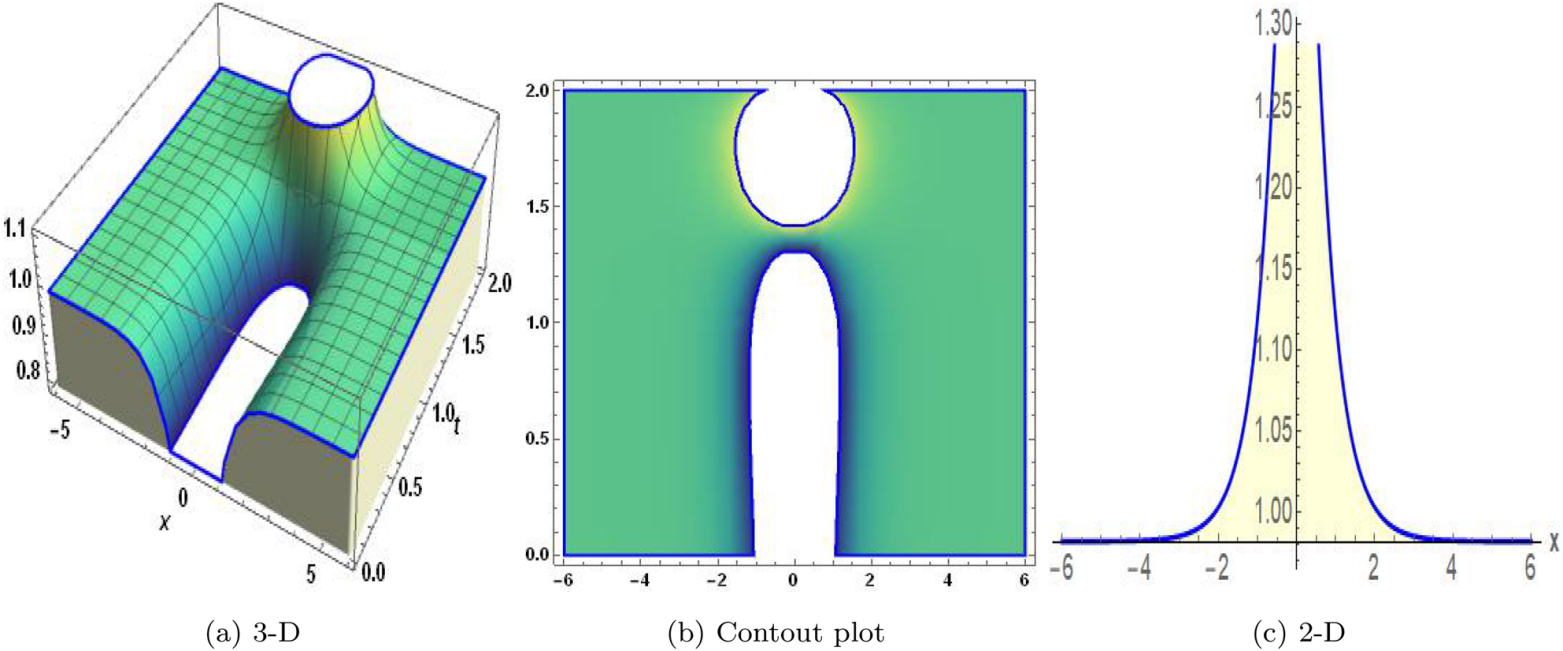
The values of parameters are $\omega _1 =1.5,~\omega _2=3.2,~b =2.03,~c=1.32,~l=1.23,~{\varsigma =0.34}~ {\text{and}} ~\Psi _{2}=1.23,$ displays the graphical representation of Eq. (52).

**Figure 6:**
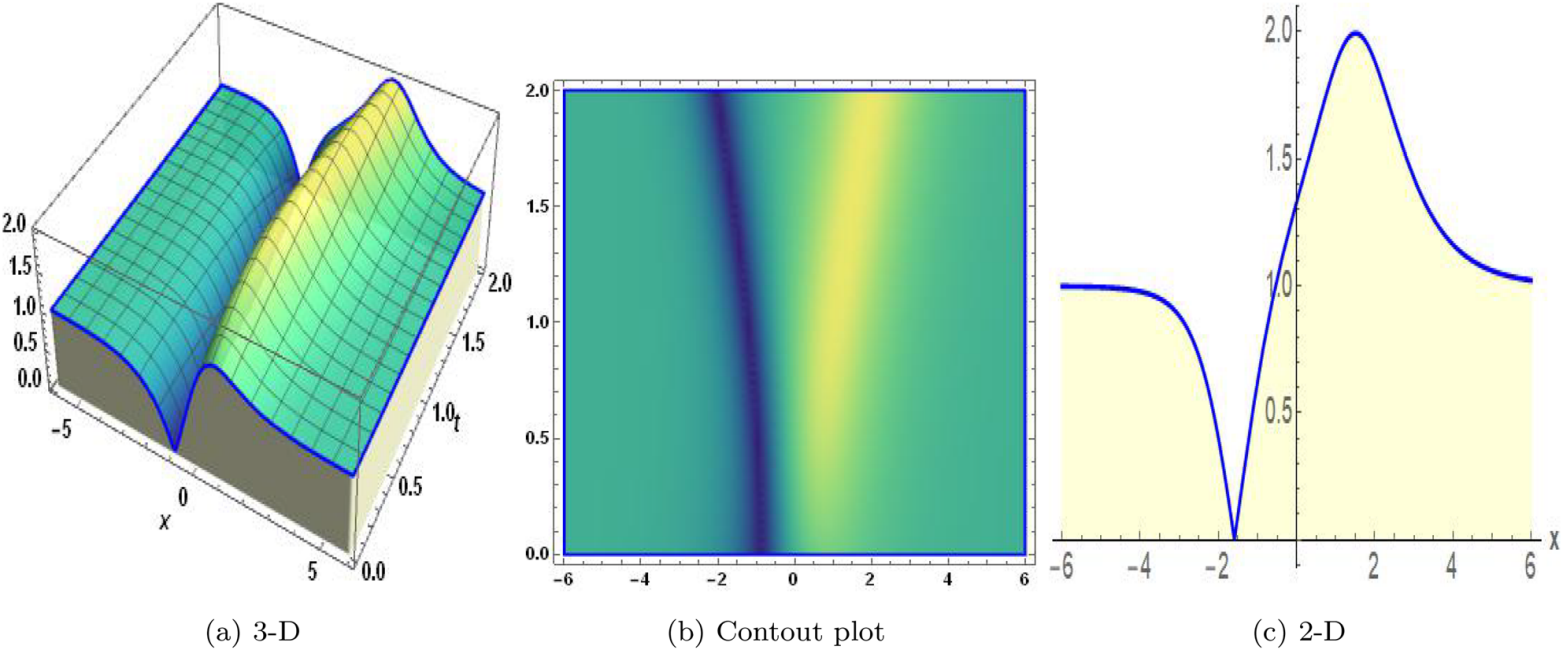
The values of parameters are $\omega _1 =1.5,~\omega _2=3.2,~b =2.03,~c=1.32,~l=1.23,~{\varsigma =0.9}~ {\text{and}} ~\Psi _{2}=1.23,$ displays the graphical representation of Eq. (55).

**Figure 7:**
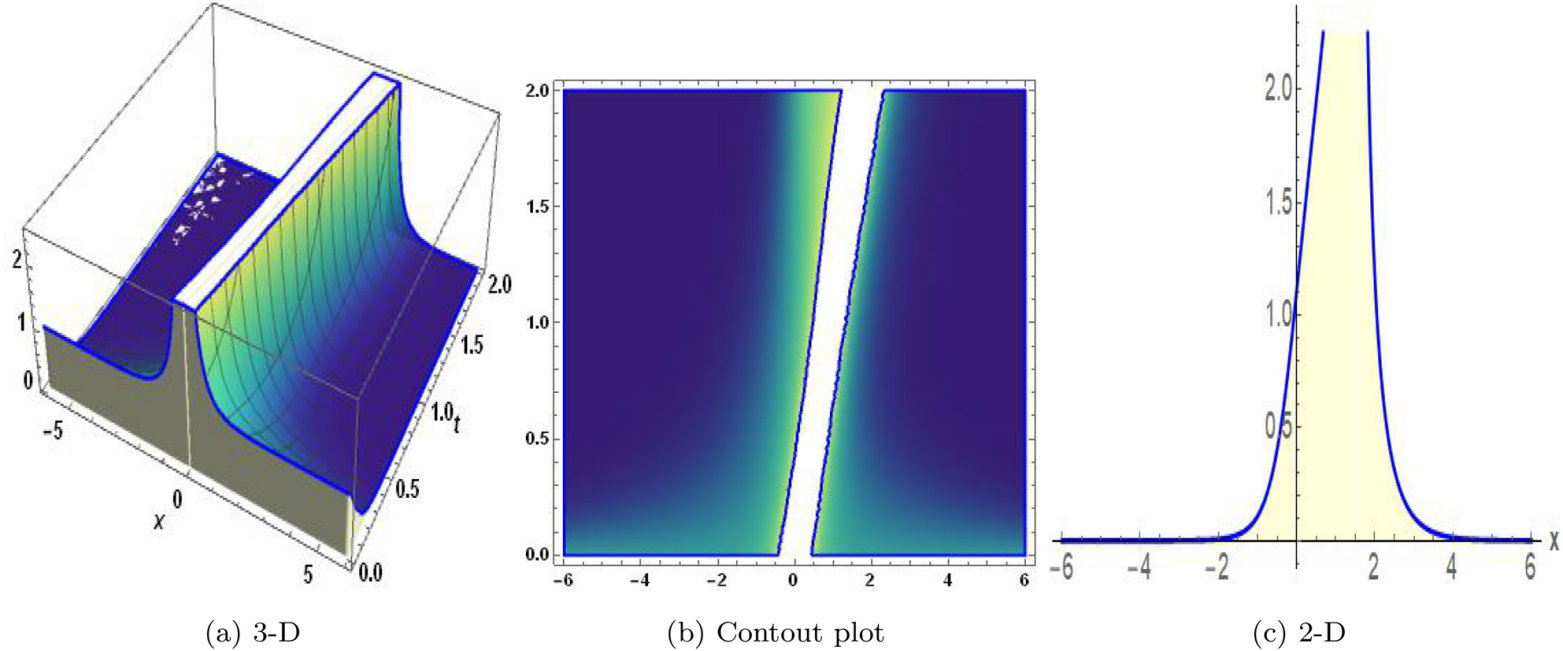
The values of parameters are $\omega _1 =1.5,~\omega _2=3.2,~b =2.03,~c=1.32,~l=1.23,~{\varsigma =1.94}~ {\text{and}} ~\Psi _{2}=1.23,$ displays the graphical representation of Eq. (58).

**Figure 8:**
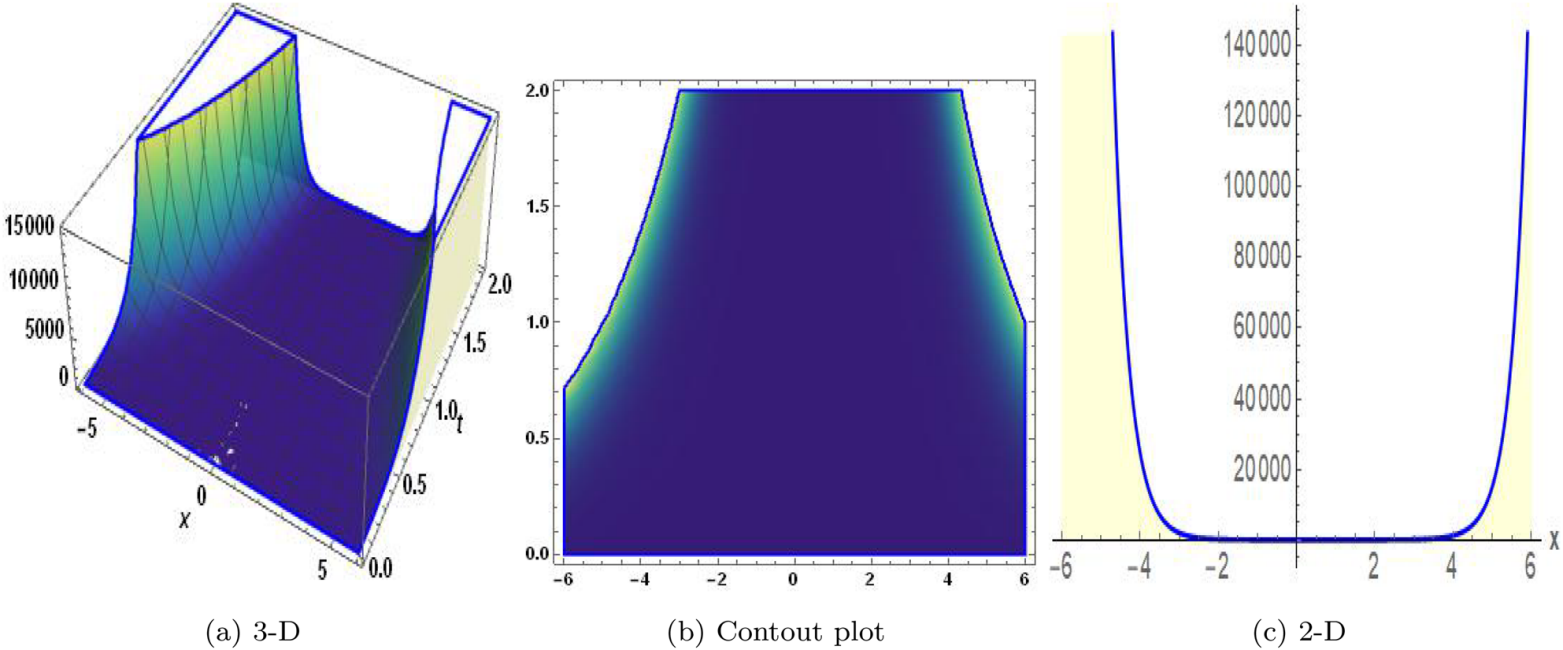
The values of parameters are $\omega _1 =1.5,~\omega _2=3.2,~b =2.03,~c=1.32,~l=1.23,~{\varsigma =0.67}~ {\text{and}} ~\Psi _{2}=1.23,$ displays the graphical representation of Eq. (61).

**Figure 9:**
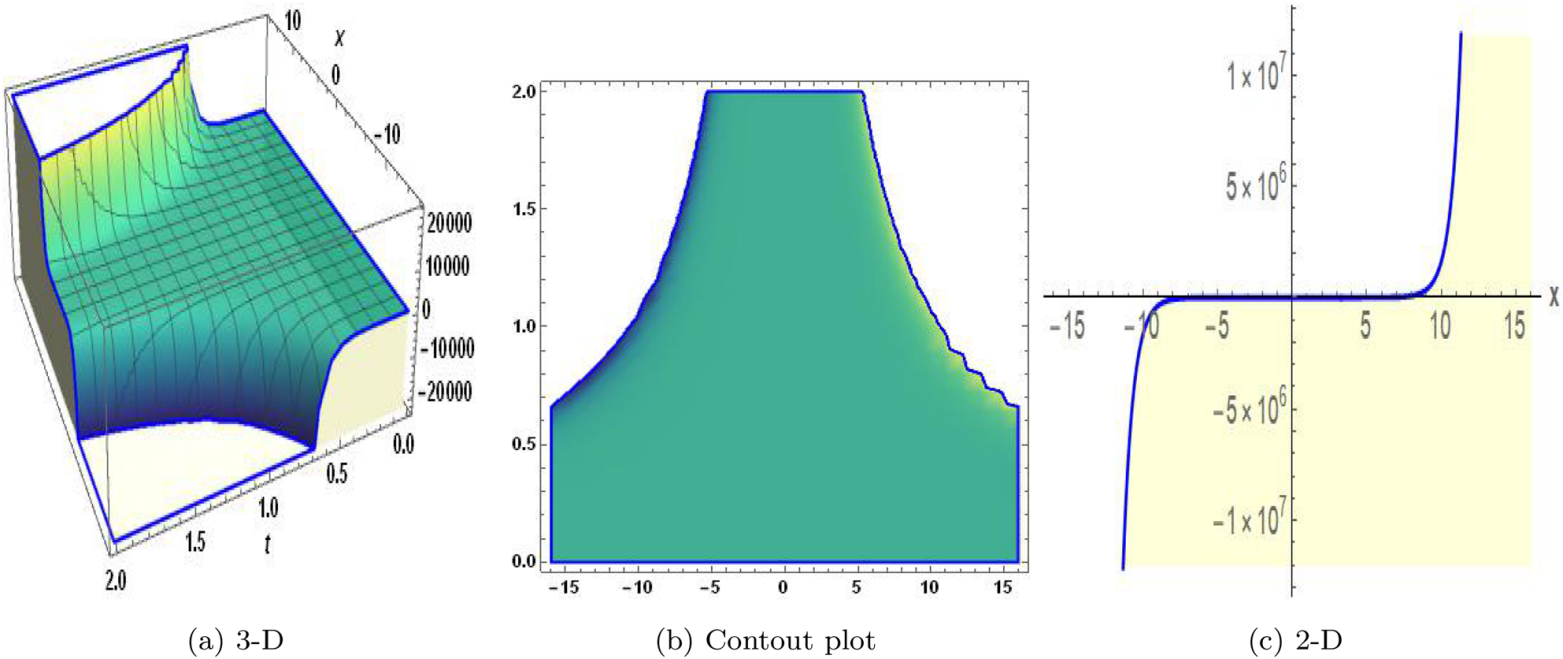
The values of parameters are $\omega _1 =1.5,~\omega _2=3.2,~b =2.03,~c=1.32,~l=1.23,~{\varsigma =1.8}~ {\text{and}} ~\Psi _{2}=1.23,$ displays the graphical representation of Eq. (64).

**Figure 10:**
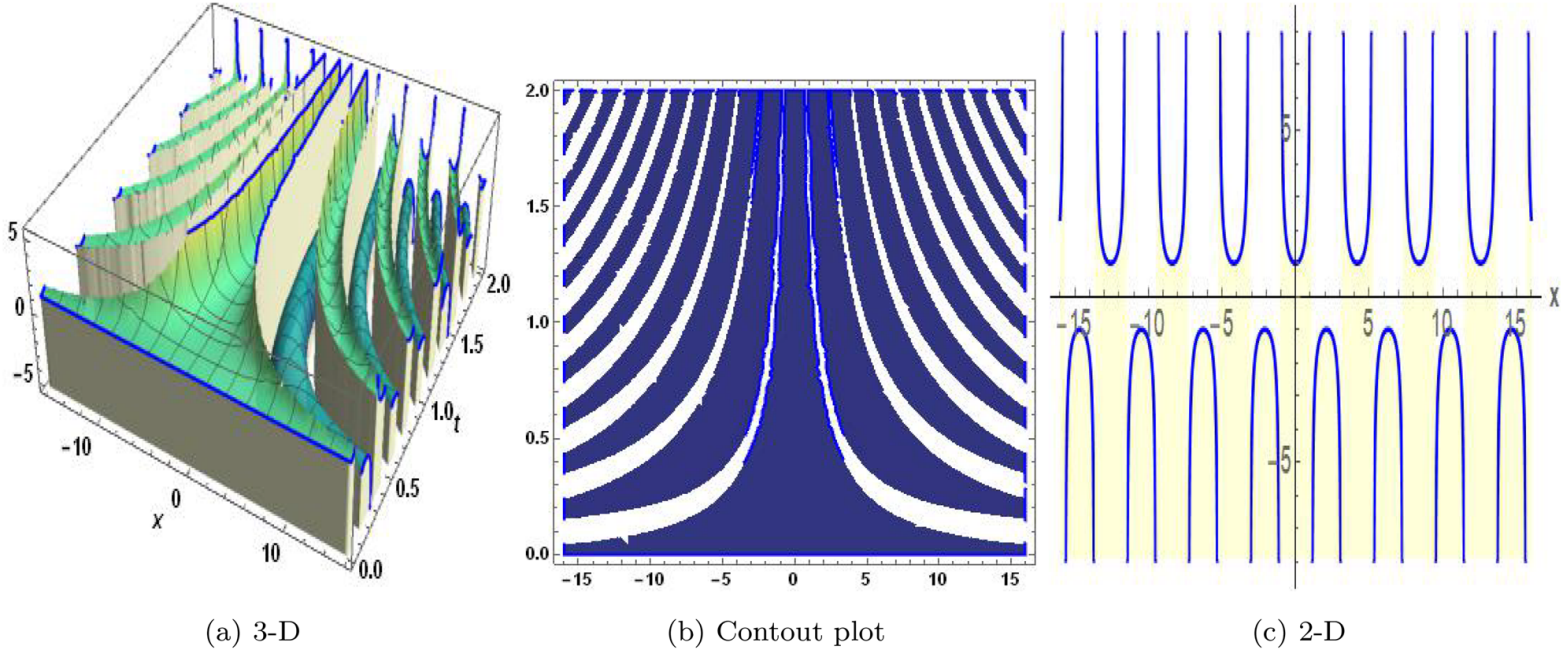
The values of parameters are $\omega _1 =1.5,~\omega _2=3.2,~b =2.03,~c=1.32,~l=1.23,~{\varsigma =0.45}~ {\text{and}} ~\Psi _{2}=1.23,$ displays the graphical representation of Eq. (67).

**Figure 11:**
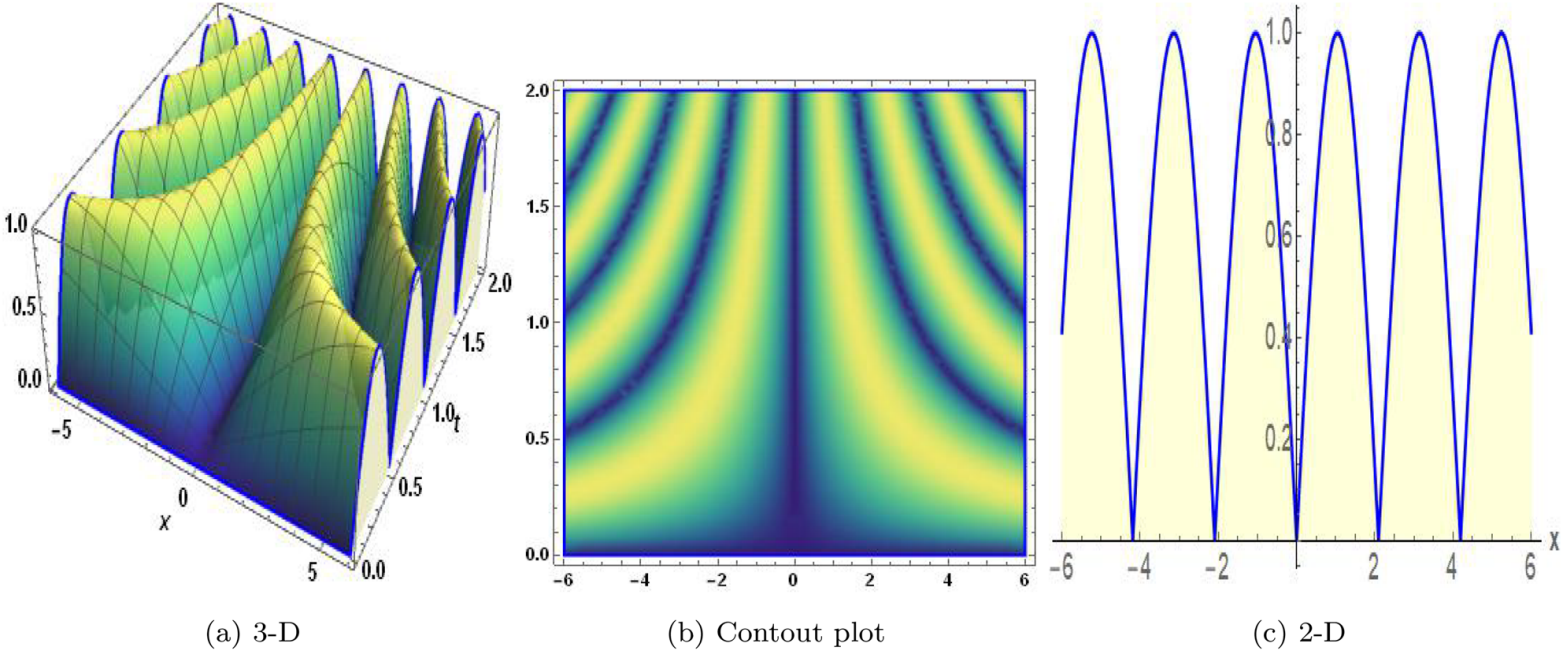
The values of parameters are $\omega _1 =1.5,~\omega _2=3.2,~b =2.03,~c=1.32,~l=1.23,~{\varsigma =0.1}~ {\text{and}} ~\Psi _{2}=1.23,$ displays the graphical representation of Eq. (70).

**Figure 12:**
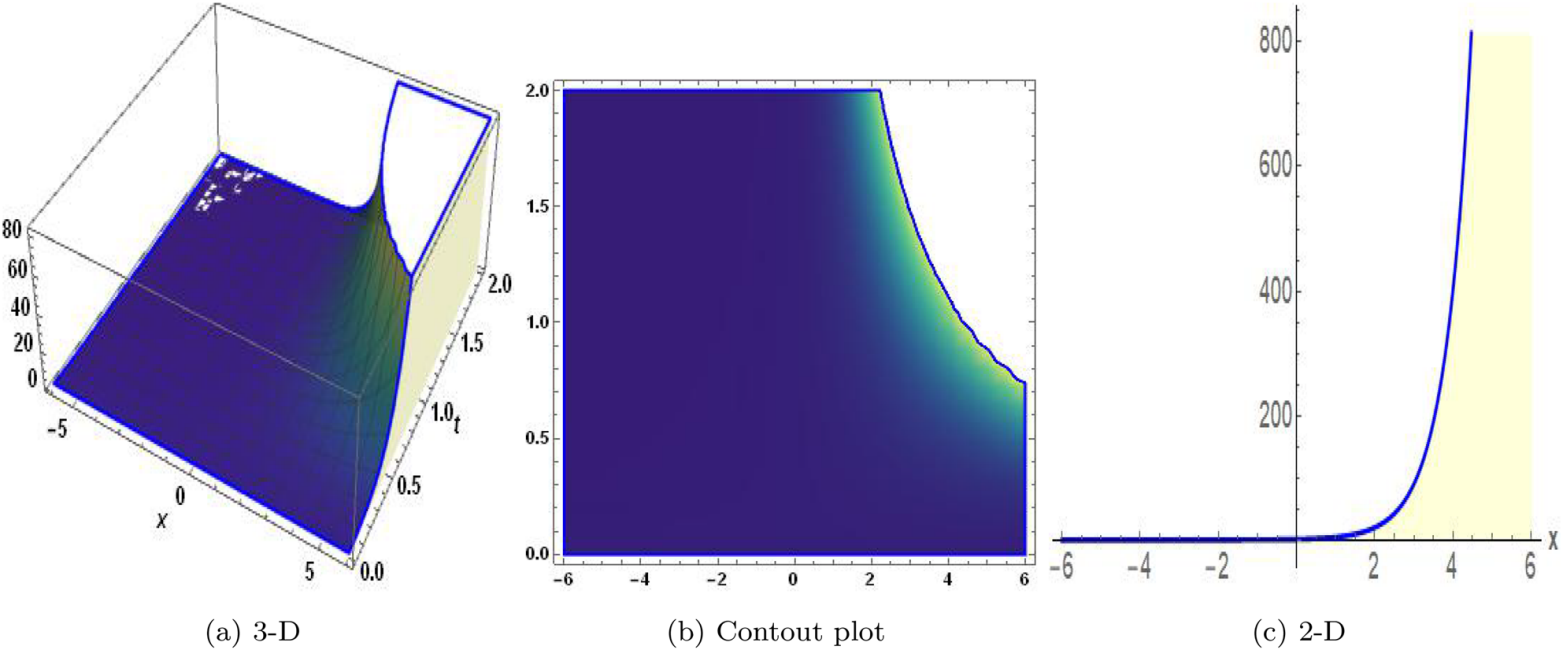
The values of parameters are $\omega _1 =1.5,~\omega _2=3.2,~b =2.03,~c=1.32,~l=1.23,~{\varsigma =0.01}~ {\text{and}} ~\Psi _{2}=1.23,$ displays the graphical representation of Eq. (95).

**Figure 13:**
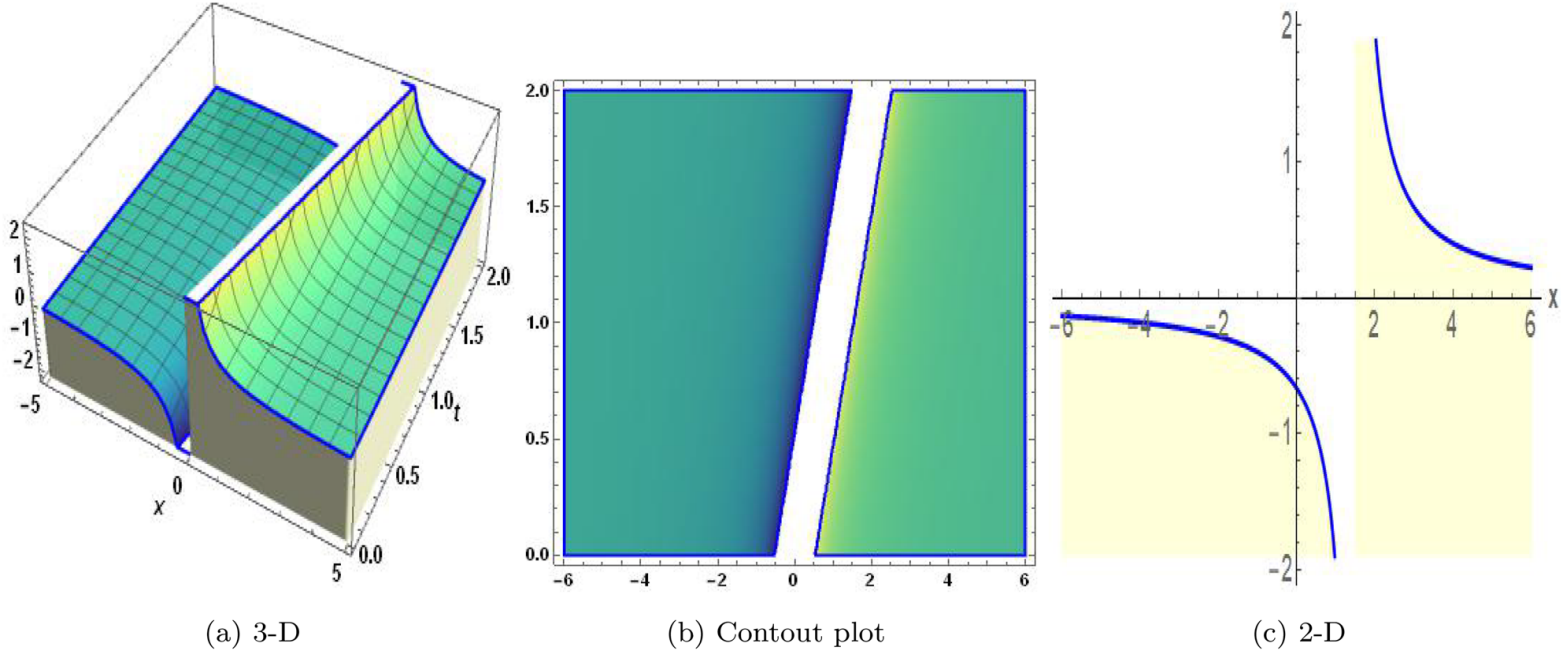
The values of parameters are $\omega _1 =1.5,~\omega _2=3.2,~b =2.03,~c=1.32,~l=1.23,~{\varsigma =0.03}~ {\text{and}} ~\Psi _{2}=1.23,$ displays the graphical representation of Eq. (98).

**Figure 14:**
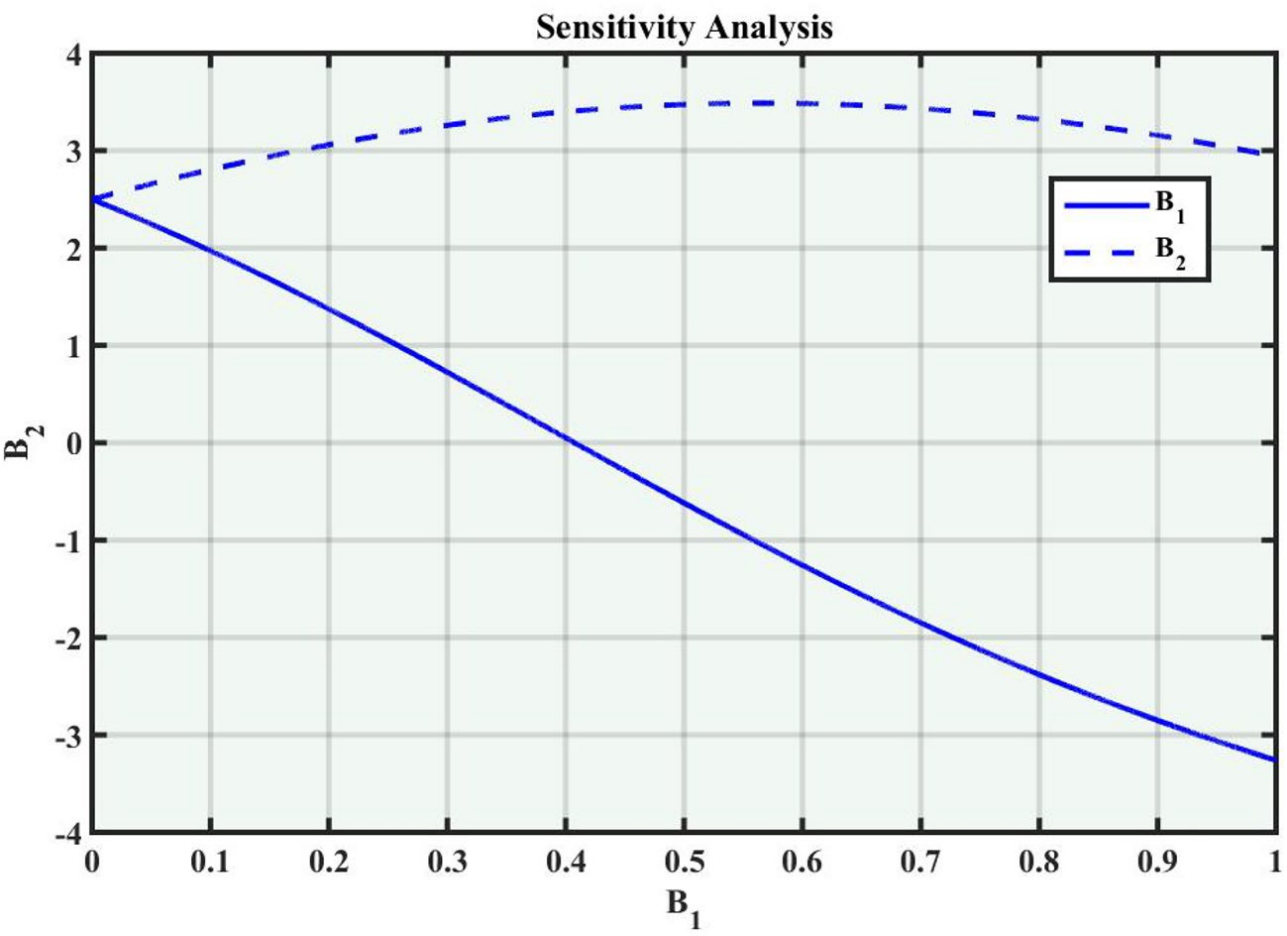
Graphical representation of Eq. (105) with condition $(B_1,~B_2)=(2.2,~2.8)$.

**Figure 15:**
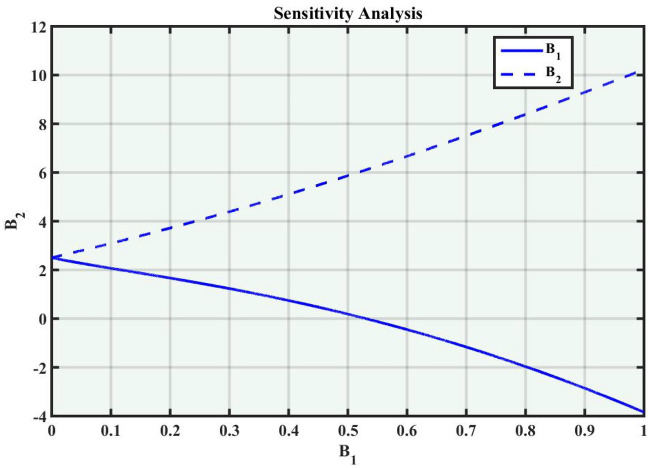
Graphical representation of Eq. (105) with condition $(B_1,~B_2)=(2.2,~2.4)$.

**Figure 16:**
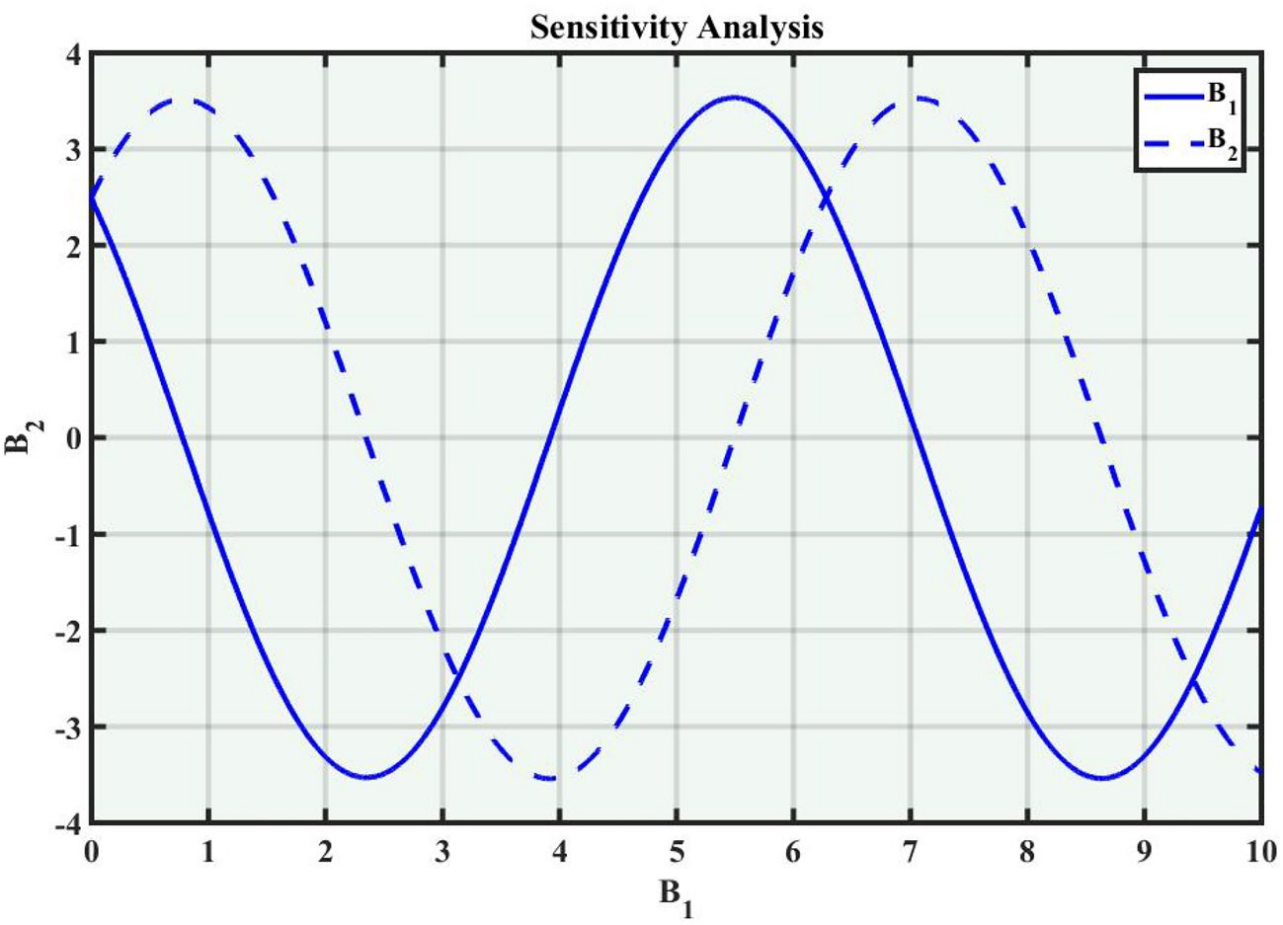
Graphical representation of Eq. (105) with condition $(B_1,~B_2)=(2.3,~2.67)$.

**Figure 17:**
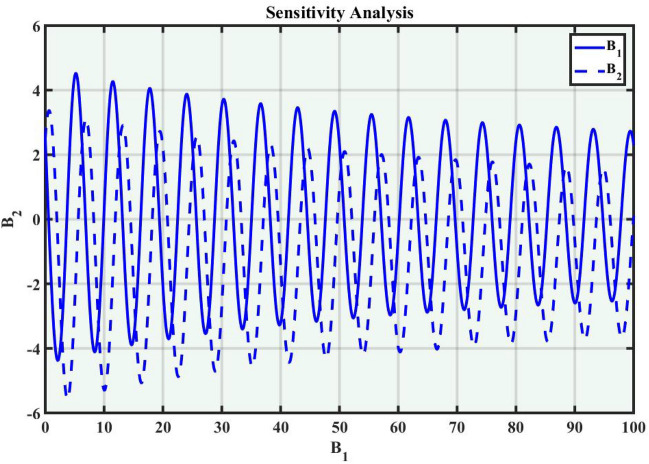
Graphical representation of Eq. (105) with condition $(B_1,~B_2)=(0.2,~0.5)$.

## Results and discussions

In this section, we compare some of our most current research findings with previous published study. Shakeel et al.^30^ employed the exponential rational approach to explore the results of time FCNLS model involving Beta derivative. In our present work, we consider (1+1)-dimensional time FCNLS model including Beta derivative and apply the MSSE approach to obtain dark, singular, periodic and rational solutions. This work presents a new technique to investigate sensitivity in model dynamics, performing time series analysis, and obtaining the optical soliton solutions. These methods work well, are simple to use, and can be applied to a variety of complex systems. Earlier research, on the other hand, was mainly concerned with determining optical soliton solutions and investigating sensitivity in model behavior. We build on this in our research by adding time series analysis, which offers a thorough comprehension of the dynamic behavior of the model. In addition, our findings provide new perspectives on how to use MSSE to investigate sensitivity and obtain soliton solutions, which advances the field of nonlinear dynamics studies. Localised areas of a wave’s lower intensity are represented by dark solitons. In physical systems such as optical fibers, they correspond to regions of minimum light intensity, frequently as a result of dispersion being counteracted by nonlinear processes. Localized intensified patches within a wave are the defining feature of bright solitons. Within optical systems, they represent regions of high light intensity, usually due to nonlinear effects counteracting dispersion. Within a wave, sudden changes or areas of severe behavior are indicated by singular soliton solutions. The study of wave dynamics, including electromagnetic wave propagation, depends on these solutions, which can be found in many different physical processes. Insights into wave behavior that can be characterized by straightforward mathematical relationships are provided by rational soliton solutions, which are wave patterns controlled by rational functions. The framework of obtained solitons is depicted in Figs. 1, 2, 3, 4, 5, 6, 7,8, 9, 10, 11, 12 and 13 and every feasible portrait of sensitivity analysis is explored in Figs. 14, 15, 16 and 17.

A single wave with singular soliton solutions shows that derivatives are discontinuous. Compactions and peakons having peaks with discontinuous first derivatives, are two examples. Periodic solutions are very important in various branches of technology because they occur again over time. Rational approaches are very beneficial in mathematics subjects including geometry, calculus, and numerical methods. These methods help in pattern recognition, connecting various sorts of solutions, and offering insights on equation structures. This technique may be constrained by its limited applicability to particular equation types or issue domains. The efficiency of the approach depends on constraint relations on parameters, which aren’t always easily met or appropriate in every situation. Controlling the method’s complexity, especially when working with large equations or systems, may provide difficulties and reduce its effectiveness. Although the method yields analytical answers, it might not always provide great precision, particularly for highly nonlinear or complicated systems, which could result in errors. Even with these benefits, there might still be opportunities for algorithmic refinements or greater generalizability to a larger class of equations and issues.

### Graphical description

Fig. (1) demonstrates bright soliton solutions of Eq. (41). Fig. (2) explores singular soliton solutions of Eq. (44). Fig. (3) illustrates kink-type solutions of Eq. (47). Fig. (4) shows dark solutions of Eq. (52). Fig. (5) represents singular solutions of Eq. (58). Fig. (6), illustrates the combo soliton solutions of Eq. (55). Fig. (7), illustrates the singular solutions of Eq. (58). Fig. (8), illustrates the U-shaped singular dark solutions of Eq. (61). Fig. (9) demonstrates bright singular solutions of Eq. (64). Fig. (10) explores periodic solutions of Eq. (67). Fig. (11), illustrates the combo of periodic solutions of Eq. (70). Fig. (12) shows the exponential solutions of Eq. (95). Fig. (13) represents rational solutions of Eq. (98). Figs. 14, 15, 16 and 17, illustrates the physical depiction of sensitivity analysis.

## Conclusion remarks

This work explores a (1+1)-dimensional temporal FCNLS model for fibre optic wave analysis that includes Beta fractional derivatives. We extract soliton solutions and perform a qualitative model evaluation using the MSSE approach. The solutions have been found in single, periodic, combination, dark, and rational solutions. Using sensitivity analysis, we investigate the sensitivity of the dynamical system and expose its dependency on several physical parameters with novel insights. These techniques provide a dynamic mathematical tool for solving a variety of nonlinear wave difficulties in mathematical physics, engineering, fibre optic waves, and other nonlinear domains. These results may be important for comprehending how fibre optic waves spread in oceanography.

## Data Availability

All data generated or analysed during this study are included in this published article.
